# Positional motif analysis reveals the extent of specificity of protein-RNA interactions observed by CLIP

**DOI:** 10.1186/s13059-022-02755-2

**Published:** 2022-09-09

**Authors:** Klara Kuret, Aram Gustav Amalietti, D. Marc Jones, Charlotte Capitanchik, Jernej Ule

**Affiliations:** 1 National Institute of Chemistry, Hajdrihova 19, SI-1001 Ljubljana, Slovenia; 2 Jozef Stefan International Postgraduate School, Jamova cesta 39, 1000 Ljubljana, Slovenia; 3 The Francis Crick Institute, 1 Midland Road, London, NW1 1AT UK; 4 UK Dementia Research Institute, King’s College London, London, UK

**Keywords:** RNA-binding protein, Protein-RNA interaction, CLIP, k-mer, RNA-binding specificity, RNA motif, Low-complexity region

## Abstract

**Background:**

Crosslinking and immunoprecipitation (CLIP) is a method used to identify in vivo RNA–protein binding sites on a transcriptome-wide scale. With the increasing amounts of available data for RNA-binding proteins (RBPs), it is important to understand to what degree the enriched motifs specify the RNA-binding profiles of RBPs in cells.

**Results:**

We develop positionally enriched k-mer analysis (PEKA), a computational tool for efficient analysis of enriched motifs from individual CLIP datasets, which minimizes the impact of technical and regional genomic biases by internal data normalization. We cross-validate PEKA with mCross and show that the use of input control for background correction is not required to yield high specificity of enriched motifs. We identify motif classes with common enrichment patterns across eCLIP datasets and across RNA regions, while also observing variations in the specificity and the extent of motif enrichment across eCLIP datasets, between variant CLIP protocols, and between CLIP and in vitro binding data. Thereby, we gain insights into the contributions of technical and regional genomic biases to the enriched motifs, and find how motif enrichment features relate to the domain composition and low-complexity regions of the studied proteins.

**Conclusions:**

Our study provides insights into the overall contributions of regional binding preferences, protein domains, and low-complexity regions to the specificity of protein-RNA interactions, and shows the value of cross-motif and cross-RBP comparison for data interpretation. Our results are presented for exploratory analysis via an online platform in an RBP-centric and motif-centric manner (https://imaps.goodwright.com/apps/peka/).

**Supplementary Information:**

The online version contains supplementary material available at 10.1186/s13059-022-02755-2.

## Background

RNA regulation is mediated through dynamic interactions with RNA-binding proteins (RBPs) [1]. To understand how RBP-RNA interactions direct cellular processes, it is first necessary to identify RBP binding sites on the RNA, which is commonly achieved with crosslinking and immunoprecipitation (CLIP) technologies [2]. To date, the largest resource of CLIP data has been produced using the eCLIP method on 150 RBPs by the ENCODE consortium [3–5], thus enabling systematic studies of the features that recruit RBPs to specific RNA sites. While these data have been used to identify the sequence motifs bound by RBPs, the extent of specificity and enrichment of these motifs has not yet been compared across RBPs. Such analysis could inform on the extent to which RNA sequence determines the specificity of protein-RNA interactions, as well as on the quality and specificity of the CLIP data itself.

CLIP uses UV light to crosslink the direct protein-RNA contacts, followed by recovery of crosslinked RNA fragments to obtain a transcriptome-wide crosslinking landscape of a specific protein [6]. After crosslinking, the cells are lysed, RNA is partially fragmented, and the selected RBP is purified along with the crosslinked RNA fragments, which are used to prepare cDNA libraries for high-throughput sequencing. Resulting sequencing data is processed initially to identify the genomic positions of the nucleotides that crosslinked to the studied RBP and then leverage these positions to obtain regions with high density of RBP crosslinking, termed peaks. Subsequently, peaks are used to derive features driving the specificity of RBP binding, such as RNA sequence motifs or RNA structural elements.

Many methods have been developed for motif discovery from CLIP data, which employ various strategies to modelling and encode input features with different levels of complexity [7, 8]. Some of the approaches transcend motif discovery from individual CLIP datasets and apply machine learning for comparative analyses of dozens of RBPs at once; however, the applicability of such methods depends on availability of large CLIP resources with low technical variations or batch effects between datasets [9, 10]. Conversely, algorithms that identify biologically relevant motifs from individual CLIP datasets usually require only a set of foreground and background sequences extracted from the CLIP data. For example, the mCross method derives foreground sequences from crosslinking peaks and background sequences from flanking regions upstream (-550…-450nt) and downstream (450...550nt) from the peak center [11]. Similarly, the ENCODE study derived foreground sequences from eCLIP peaks and background sequences from random areas within the same gene matched for length and transcript region [3, 12].

Analysis of crosslink-associated features, as compared to full sequencing reads, increases the accuracy of tools that identify enriched motifs [11, 13], because crosslink sites tend to coincide or be in close proximity of the RNA sequences that are recognized by the RBP. Nevertheless, motifs enriched at crosslink sites can also reflect technical biases of CLIP experiments, such as the sequence and structural preferences of UV crosslinking [6], as well as the preferences of enzymes used for RNA fragmentation, ligation and reverse transcription [14], and sequence variations between genomic regions bound by different RBPs. The impact of these potential biases on motifs identified from CLIP data is poorly understood due to the lack of systematic studies [6].

Here we developed positionally enriched k-mer analysis (PEKA), a computational method for detailed examination of RBP’s binding preferences. PEKA enables discovery and visualization of enriched motif clusters for various RNA regions (introns, 3′-UTR, ncRNA etc.), which can be performed with or without considering the repetitive elements. To account for crosslinking biases and other technical features that affect the starts of reads in cDNA truncation-based methods (iCLIP and related) and transitions in PAR-CLIP [6], PEKA uses low-count crosslinks from the analyzed dataset as a background. We show that PEKA performs equally well as mCross k-mer analysis when benchmarked using orthogonal in vitro data, while also allowing more flexible analysis tailored to the studied RBP. Here, we employ PEKA to perform comparative analysis of motif enrichment across 150 RBPs, using 223 publicly available eCLIP datasets, 59 PAR-CLIP datasets, and 4 iCLIP datasets. We integrate it with orthogonal in vitro data to examine technical biases of eCLIP and PAR-CLIP and to show that the extent of motif enrichment and the presence of commonly enriched motifs are useful signatures of data quality, and are linked to the domain composition, crosslinking efficiencies, and regional binding preferences of the studied RBPs.

By being able to derive reliable enriched motifs from analysis of individual CLIP datasets, PEKA will be useful to those performing CLIP experiments of any scale, and in any cell type or species. PEKA is licensed under an open-source GNU General Public License v3.0 and can be easily installed from GitHub [15] or Bioconda [16]. Furthermore, the software is integrated into the iMAPS webserver [17], where it can be used for interactive, reproducible, and password-protected analysis of uploaded CLIP data. Moreover, we provide interactive analyses of eCLIP data in a variety of formats via a web interface [18], including a protein-centric view of motif enrichments and profiles around crosslinks of specific RBPs, and a motif-centric analysis of comparative motif distributions around crosslinks of multiple RBPs. Such user-friendly discovery and exploratory visualization of RNA-binding motifs is a crucial step towards identifying the functionally relevant binding sites from CLIP data as a basis for further studies.

## Results

### Positionally enriched k-mer analysis (PEKA) overview

**Fig. 1:**
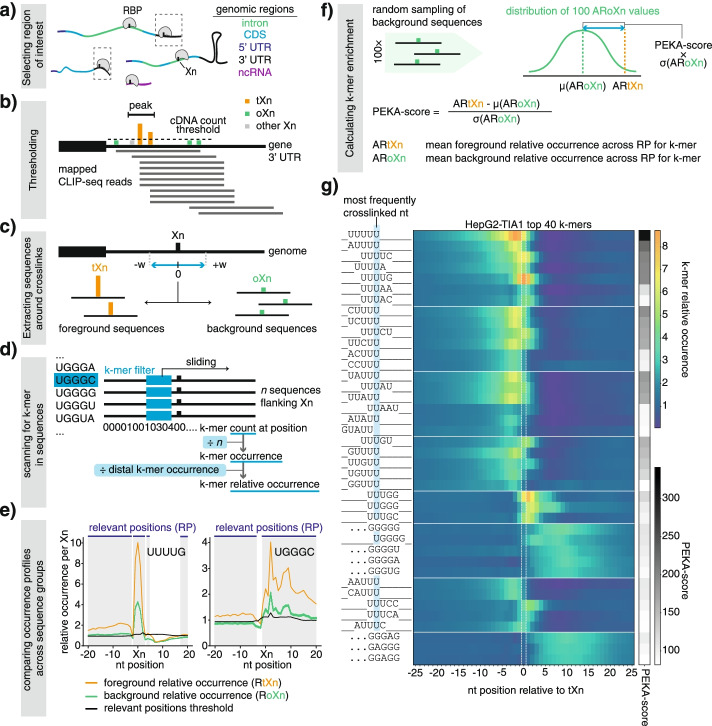
Schematic representation of the positionally enriched k-mer analysis (PEKA). **a** Most eCLIP datasets have sufficient coverage on multiple transcriptomic regions to enable each to be investigated separately for motif enrichment by PEKA. **b** Two bed files with the location of putative crosslink sites (Xn) and peaks are provided as inputs, so that PEKA can separate the Xn into the thresholded (tXn) and reference crosslink sites (oXn). Crosslinks that are not classified as tXn or oXn are marked as other Xn. tXn lie within peaks and have a cDNA count above a regional threshold (can be modulated by the user, see “Methods” for details), while oXn lie outside of peaks. **c** Foreground and background sequences flanking tXn and oXn, respectively, are retrieved. The width (w) of the flanking region can be specified by the user. **d** Sequences are scanned to record whether or not a k-mer is present at a particular position. For each group of sequences, k-mer occurrence around Xn is calculated and then converted to relative occurrence by dividing by mean k-mer occurrence across the distal window located 100 nt upstream and downstream from tXn (distal k-mer occurrence). **e** Positions around Xn where foreground relative occurrence passes the “position-threshold value” are considered as “relevant positions” for enrichment analysis of each k-mer. **f** PEKA score is calculated for each k-mer by comparing the mean k-mer relative occurrence across the relevant positions around tXn vs randomly sampled oXn, and used to rank the k-mers. **g** Heatmap showing relative occurrences (RtXn) and PEKA scores for top 40 k-mers identified by PEKA for TIA1 eCLIP in HepG2 cell line. K-mers are clustered based on their sequences, which are aligned to the position of their maximal RtXn (on the left), and the most frequently crosslinked nucleotide is highlighted in blue. When the maximal occurrence position is located further than 3 nt from the crosslink site, three dots are shown

PEKA includes several features that are designed to examine and minimize the impact of technical biases of CLIP, and thereby to obtain enriched motifs that mediate RBP binding specificity. PEKA can perform motif discovery either across the full transcriptome or within defined transcriptomic regions, with the provided options including introns, 3′-UTR, remaining exonic regions of protein-coding genes (coding sequence (CDS) combined with 5′-UTR), non-coding RNAs (ncRNAs), and the rest of non-annotated intergenic regions (Fig. 1a). PEKA also provides an option to include or exclude repetitive regions in the analysis.

Importantly, PEKA implements an approach of background normalization that aims to minimize the technical biases at crosslink sites. Crosslink sites are determined by the first nucleotide of aligned sequencing reads, which can include nucleotide preferences of UV crosslinking or sequence biases of cDNA ligation [6, 14]. To normalize for these biases, PEKA extracts the background sequences that are centered on low-scoring crosslink sites (out-of-peak crosslinks, oXn), which are located outside the peaks (i.e., areas with high crosslink density). The peak file is provided by the user, and peaks can be identified by any peak calling tool. For the current study, peaks have been identified with our newly developed peak caller Clippy [19] (“Methods”) due to its short runtime and the ability to call peaks in an annotation-aware manner, to correct for biases of highly expressed genes and exons. Conversely, the foreground sequences are centered on high-scoring crosslink sites located inside the crosslinking peaks (thresholded crosslinks, tXn). The first step of PEKA is thus to split crosslink sites into thresholded (tXn) and out-of-peak (oXn) sites based on whether the sites overlap with a peak and whether the cDNA count at the site meets a minimum threshold that is defined in a region-specific manner (Fig. 1b, “Methods”). Notably, tXn and oXn sites are expected to be affected by the same technical biases since they are part of the same cDNA library.

PEKA derives background and foreground sequences from user-defined genomic windows centered on tXn and oXn, respectively (Fig. 1c). In the current study, a distance of 20 nt was used to define proximal windows used to collect the sequences (41 nt long, i.e., ±20 nt distance from Xn). The script allows users to adjust the size of the proximal window if they wish to narrow down the search for motifs closer or expand it further away from the crosslink sites. By scanning for the presence of k-mers across sequences, PEKA obtains k-mer counts at each sequence position and then transforms them into k-mer occurrences by normalizing with the number of evaluated sequences (Fig. 1d). Next, the “relevant positions” around crosslink sites are defined at which the enrichment is calculated for each k-mer (Fig. 1e). For this purpose, “relative k-mer occurrence” is calculated by normalizing occurrence at each proximal position with the mean occurrence in a distal window (defined as −150…−100 and 100…150 nt around tXn). Relevant positions are the ones where “relative k-mer occurrence” of each specific k-mer around tXn is higher than a threshold value that is determined based on relative occurrences of all k-mers (see “Methods” for more detail, Fig. 1d). The relevant positions are then used to calculate a PEKA score that represents motif enrichment. Alternatively, PEKA also offers users an option to calculate enrichment by using all positions within a proximal window without the process of selecting the “relevant positions.”

PEKA score conveys the extent of k-mer enrichment at relevant positions around thresholded crosslink sites (tXn) relative to the same relevant positions around out-of-peak crosslink sites (oXn), which represent the intrinsic background of the studied dataset (Fig. 1e,f). PEKA score is a derivative of standard score and measures the number of standard deviations separating the approximated motif occurrence around tXn (ARtXn) from the mean approximated occurrence around oXn (μ(ARoXn)) (Fig. 1f, “Methods”). For further analyses, the motifs are ranked based on PEKA score in a descending order and the top *n* k-mers are selected to visualize their positioning around tXn with occurrence profiles. By default, PEKA separates the top 20 k-mers into up to 5 clusters based on their similarity in sequence and occurrence profiles, and then visualizes the profiles of k-mers within each cluster on the same graph (Additional file 1: Fig. S1a). For heatmap visualizations, the top 40 k-mers are clustered based on their sequence, and their relative occurrence profiles are shown (Fig. 1g). The use of relative occurrence, which normalizes the raw k-mer occurrences with distal window (Fig. 1d), improves the capacity to compare the enriched positions of different k-mers, as otherwise the regional genomic differences of k-mers would decrease the visibility of the less-abundant k-mers, as shown by comparing the two approaches for LIN28B (Additional file 1: Fig. S2a,d).

### PEKA detects multiple binding modes of RBPs

To get preliminary insights into the distribution and sequence characteristics of the enriched k-mers around crosslink sites, we first analyzed the occurrence profiles of 5-mers for the well-studied protein TIA1 in eCLIP (Fig. 1g). The 5-mer profiles for TIA1 eCLIP in the HepG2 cell line show the most enriched motifs to be U-rich (Fig. 1g), in agreement with the known sequence specificity of TIA proteins [20]. In particular, the top 18 k-mers are all U-rich, followed by “GGGGG” and several other G-rich k-mers. Interestingly, U-rich motifs are enriched directly at crosslink sites, whereas G-rich motifs are depleted at crosslink sites and enriched primarily downstream of the crosslink sites. These non-overlapping positional patterns of the two groups of motifs indicate either that they represent different modes of binding by TIA1, or that the G-rich motifs are derived from other co-purified RBPs that bind to the G-rich motifs. To examine whether G-rich k-mers indeed represent a novel binding mode of TIA1, we examined whether the enrichment of these k-mers could be reproduced by other CLIP methods. For this, we compared occurrence profiles of enriched k-mers for TIA1 iCLIP and TIA1 PAR-CLIP and found that enrichment of G-rich k-mers was specific to eCLIP (Additional file 1: Fig. S1b,c). This case study of TIA1 data from three different CLIP methods demonstrates that k-mers with similar sequences tend to have similar positional profiles around crosslink sites, the applicability of PEKA to various CLIP datasets, and the ability of PEKA to report multiple motif types with distinct profiles, which can yield insights into data specificity or multiple binding modes of RBPs.

To corroborate the ability of PEKA to detect multiple binding modes of RBPs, we plotted positional profiles of enriched motifs for two RBPs that are known to bind several types of motifs (Additional file 1: Fig. S2a-c). Indeed, the top 20 k-mers for TARDBP eCLIP form three groups (UG-repeat, GUAU-motifs, and UGAA-motifs) which are consistent with those previously found to have distinct binding preferences to mutant variants of TARDBP in iCLIP experiments [21]. Moreover, LIN28 is known to bind (U)GAU k-mer, as well as GGAG-like sequences [22], which are also both recovered among the top 20 enriched k-mers in eCLIP data. Finally, PEKA is able to detect and visualize complex binding patterns, such as bipartite motifs bound by QKI [23, 24] which are not apparent from the PWM-based visualizations provided by other methods for motif discovery. Given the useful insights derived from this visualization, we provide heatmaps of the top 40 k-mers determined by PEKA for all 223 ENCODE eCLIP datasets on the web interface we developed to share our results with the community [18].

### Benchmarking of PEKA against mCross and in vitro data

A major challenge in CLIP data analysis is the identification of motifs that correspond to the RNA-binding specificity of the isolated RBP, as opposed to motifs enriched for other confounding reasons, such as the technical biases of CLIP, associated features of genomic regions and repetitive elements bound by the protein, or motifs bound by other RBPs that may be co-purified if IP stringency was insufficient [6]. To evaluate the specificity of enriched motifs, these can be cross-compared with the motifs obtained by in vitro methods, in particular the high-throughput methods RNA-Bind-n-Seq (RBNS) [25] and RNAcompete (RNAC) [26]. In both RNAC and RBNS, a specific RBP or an assembly of its RNA-binding domains is mixed with a random pool of RNAs, followed by the isolation and sequencing of bound RNA molecules. These in vitro methods are expected to have different biases than CLIP data, and their limitations arise mainly from the use of short RNA fragments and the absence of full-length proteins and their cofactors. Thus, data produced with these methods is well suited to examine the biological specificity of motifs derived from CLIP data.

To examine the capacity of PEKA to recognize the top-ranking motifs obtained from in vitro data, we compared the top 20 k-mers recovered from in vitro RBNS/RNAC datasets with the motifs identified by PEKA in the corresponding eCLIP data (Additional file 1: Fig. S3a). We also compared the performance of PEKA with that of a previously published mCross method on the same data [11]. In total, 41 eCLIP datasets (Additional file 2: Table S1) were compared for 28 distinct proteins as they contain both mCross and in vitro data (Additional file 1: Fig. S3a). In addition, we analyzed the recall achieved by PEKA in eCLIP datasets which have orthogonal in vitro data available but did not yield significant motif enrichment in mCross (Additional file 1: Fig. S3b). As RBNS provides 5-mer scores, PEKA was also run for 5-mers, and to ensure a valid comparison, the mCross and RNAC *z*-scores were converted from 7-mer to 5-mer scores (“Methods”) prior to ranking the motifs. Ranking k-mers by normalized mCross 5-mer z-scores resulted in k-mer logos (Additional file 1: Fig. S3c) that were very similar to the mCross sequence logos downloaded from the mCrossBase [11, 27] (Additional file 1: Fig. S3d), confirming that the relevant k-mers were retained during this conversion. Importantly, PEKA overall showed a similar performance to mCross in recovering the top 20 k-mers from in vitro data (Additional file 1: Fig. S3a, Additional file 8: Table S7) in addition to being able to achieve high recall for several datasets that did not yield significant enrichment in mCross (Additional file 1: Fig. S3b, Additional file 8: Table S7).

To compare PEKA and mCross in more detail, we returned to the G-rich motifs observed for TIA1 eCLIP in HepG2 cell line (Fig. 1g). Because these motifs are unexpected, and have not previously been reported to be bound by TIA1, they could be considered a potential artifact of PEKA. Therefore, we performed a direct comparison of the top 20 5-mers obtained for TIA1 eCLIP in HepG2 cell line, using either PEKA score, raw mCross *z*-scores, or normalized mCross *z*-scores, respectively. Based on our observation of the two k-mer groups in the data (U-rich and G-rich, Fig. 1g), the top 5-mers were divided into two clusters based on their sequence (“Methods”) and visualized as k-mer logos (Additional file 1: Fig. S3c). Reassuringly, G-rich k-mers were also detected by the raw mCross *z*-scores to a similar extent as by the PEKA scores. However, they were largely removed in the normalized mCross *z*-scores, which were obtained by relativizing the *z*-score of each k-mer in a given dataset with a distribution of *z*-scores for the same k-mer across all experiments, thereby penalizing the k-mers with high *z*-scores across many experiments [11]. PEKA does not perform any such inter-RBP normalization, and thus detection of G-rich k-mers in TIA1 eCLIP by PEKA is in line with similar enrichment seen in raw mCross *z*-scores.

### Insights from variable motif enrichments across CLIP variants

**Fig. 2:**
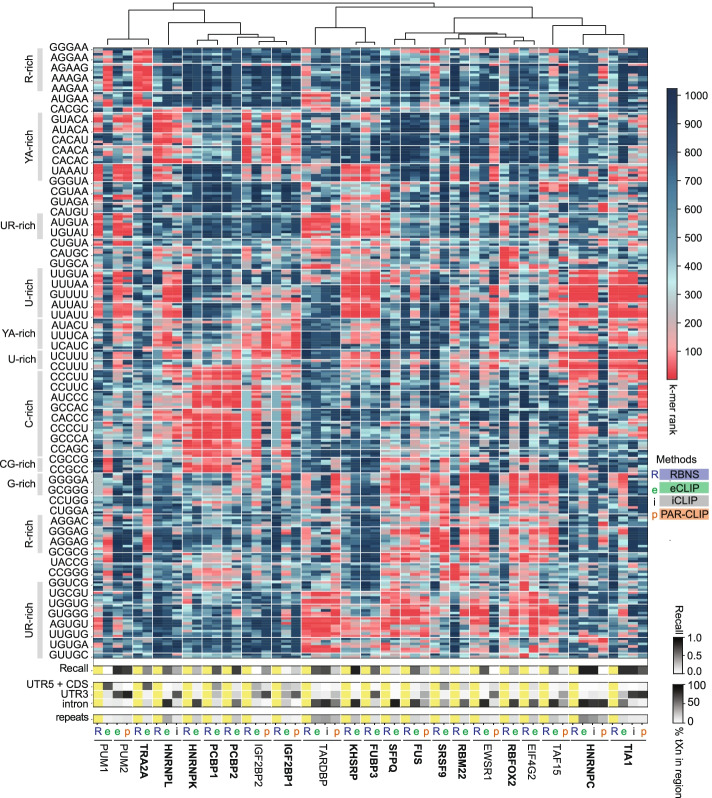
Comparison of CLIP methods against RBNS. Heatmap shows the rank order of k-mer PEKA scores for each CLIP dataset, and of RBNS *z*-scores. The scale on the top right shows these values spanning from 1 to 1024. K-mers selected for this heatmap (*n*=230) ranked among the top 10 in eCLIP, PAR-CLIP, iCLIP, or RBNS for any of the RBPs shown. K-mers were clustered based on their sequence and ordered using hierarchical clustering of median dataset ranks within clusters. On the left, prominent k-mer sequence features are highlighted across clusters. Below, the recall heatmap shows how much the top motifs in the dataset correspond with the RBNS data. The third heatmap shows the percentage of tXn derived from different transcript regions, and the fourth heatmap shows the percentage of tXn found in repeat sequences. eCLIP experiments in bold are in HepG2, others in K562 cell line. In case two eCLIP datasets for the same RBP were available, the one with higher recall is shown

To learn more about the motif preferences of different CLIP protocols, we performed a more detailed comparison of PEKA with the results obtained by in vitro method RBNS [3], which are available along with eCLIP data for 21 RBPs, iCLIP data for 4 of these RBPs, and PAR-CLIP data for 9 RBPs (Additional files 2 and 3). PEKA analysis was performed in the “protein-coding gene” region, which combines intron, CDS, and the untranslated regions (UTRs). Repeat sequences were filtered out in motif detection. Visualization of k-mer ranking across groups, generated by sequence-based clustering, forms a unique binding signature for each dataset (Fig. 2). We observed informative variations in the ranking of top k-mers between data produced by these three CLIP methods.

In the case of TARDBP, FUBP3, KHSRP, TIA1, HNRNPC, HNRNPL, PCBP1, and PCBP2, eCLIP as well as most of the available iCLIP and PAR-CLIP experiments show high agreement with RBNS (i.e., recall, Fig. 2). The G-rich motifs, which were previously observed to be enriched for TIA1 eCLIP, but not iCLIP or PAR-CLIP, are also enriched in RBFOX2 and IGF2BP1/2 eCLIP, but not in RBNS, or IGF2BP1/2 PAR-CLIP. In the case of IGF2BP1/2, additional divergence can also be attributed to the enrichment of C-rich motifs in eCLIP, which RBNS and PAR-CLIP do not exhibit. In addition to IGF2BP1/2, eCLIP experiments for PUM1, SFPQ, RBM22, and EIF4G2 also showed poor agreement with RBNS data. In the case of PUM1 and IGF2BP1/2, the data from PAR-CLIP are in much better agreement with RBNS than eCLIP. Instead of the expected motifs, G-rich motifs were enriched in the PUM1 eCLIP, suggesting that these motifs tend to be enriched when an eCLIP experiment fails to identify the expected signal (Fig. 2). Considering the known similarity of motif specificity of PUM1 and PUM2 [28], we compared the PUM2 eCLIP experiment with the in vitro data of PUM1, which showed high agreement, as reported previously [3]. This analysis demonstrates a substantial variance in the reliability of eCLIP datasets, and the value of CLIP meta-analyses to identify the datasets that are likely to be the most reliable for further studies.

We found that when CLIP datasets differ in enriched motifs, they tend to also differ in the regional distribution of crosslink sites (Fig. 2). Specifically, compared to eCLIP and iCLIP, PAR-CLIP has a generally higher proportion of tXn in the 3′-UTR relative to introns, and a lower coverage of repetitive elements. tXn distribution also varied between eCLIPs of homologous proteins PUM1 and PUM2, where we found PUM1 to have a lower proportion of tXn in the 3′-UTR and a higher proportion of tXn in CDS and 5′-UTR. Thus, a combined analysis of several CLIP features, such as motif enrichment and regional binding, may be particularly valuable for data quality assessment, and for understanding the potential generic biases of each CLIP variant.

### Peak filtering by external background yields limited benefit

**Fig. 3:**
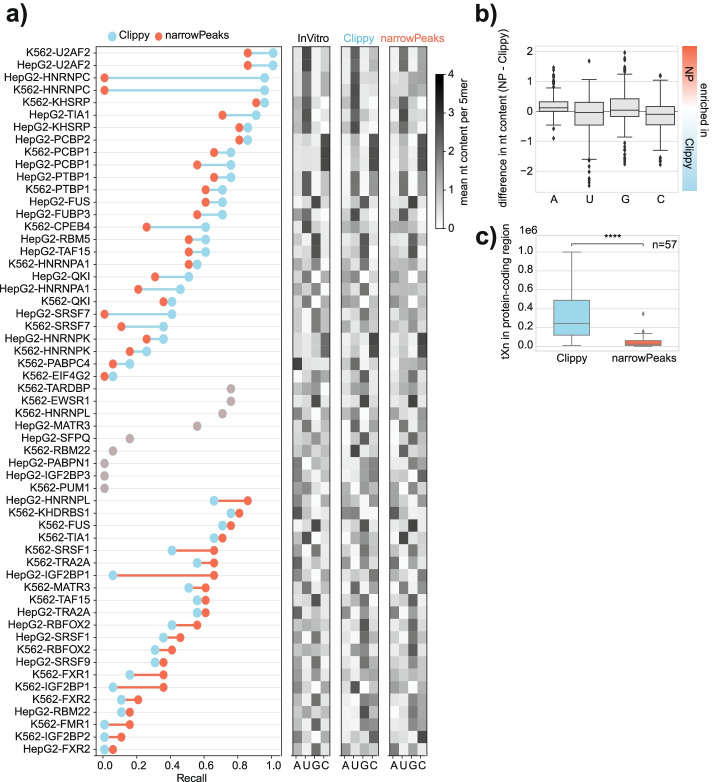
Influence of size-matched input controls on motif discovery. **a** The graph shows recall for 57 eCLIP datasets for which orthogonal RBNS or RNAcompete data was available. The datasets were processed with PEKA, using either Clippy peaks or eCLIP narrowPeaks from merged replicates, downloaded from the ENCODE consortium [4, 5]. The datasets are sorted into three groups: (1) datasets where higher recall was achieved with Clippy peaks, (2) datasets with no change in recall between groups, and (3) datasets where higher recall was achieved with narrowPeaks. Within each group, datasets are ordered by decreasing recall values. Heatmaps on the right show mean nucleotide composition across the top 50 k-mers as ranked by in vitro method, by PEKA using Clippy or PEKA using narrowPeaks. **b** Boxplots show differences in mean nt composition of top 50 k-mers as ranked by PEKA using Clippy or narrowPeaks, for 215 eCLIP datasets, which had sufficient tXn coverage in narrowPeaks for PEKA analysis. **c** Number of tXn detected by PEKA in the “protein-coding gene” region when using Clippy vs narrowPeaks (paired *t*-test, *p* < 0.0001) for 57 eCLIP datasets shown in panel **a**)

PEKA was developed for general use on any type of nucleotide-resolution genomic data, and therefore, it models motif enrichment relative to the intrinsic background of a single dataset, without the need for additional data to model extrinsic background. Nevertheless, we wished to understand if the performance of PEKA would improve if the input peaks were called taking into account the extrinsic background signal. The ENCODE consortium provides narrowPeaks, which are generated by retaining only those peaks with a significant enrichment of reads over the size-matched input (SMInput) control, which is expected to decrease the extent of extrinsic background in the data [3, 6]. Surprisingly, when we ran PEKA using only the crosslink sites located within the narrowPeaks as the foreground, we observed no overall improvement in motif specificity as compared to our Clippy peak calling approach that does not include SMInput analysis (Fig. 3a). A major increase in agreement with in vitro data when using narrowPeaks was found only for a couple of RBPs, the most prominent being IGF2BP1, but decreased agreement was seen for other RBPs, with the most prominent effect observed for hnRNPC.

We find that the approach used to derive narrowPeaks generally results in an increased adenosine content of enriched motifs (Fig. 3b), which can in turn lead to decreased content of other nucleotides, such as uridine and cytidine. As a result, the use of narrowPeaks tends to improve motif specificity for RBPs that show binding to A-rich motifs by in vitro data, but decrease specificity for proteins that bind to motifs that do not contain adenosine (Fig. 3a). To better understand this phenomenon, we visualized motif coverage of the top 20 in vitro k-mers for hnRNPC (Additional file 1: Fig. S4a) in hnRNPC eCLIP and SMInput in both cell lines. We found ~25% of cDNA start sites in SMInput have these k-mers enriched around them, in comparison to ~50% (HepG2 cells) or ~35% (K562 cells) in eCLIP (Additional file 1: Fig. S4b), with a very similar profile around crosslink sites in both cases, indicating that hnRNPC likely contributes to a major part of the foreground signal in its SMInput control (Additional file 1: Fig. S4b). Despite eCLIP having a higher proportion of these motifs enriched at cDNA start sites, this indicates that using SMInput as a control for hnRNPC eCLIP likely results in the foreground signal becoming erroneously assigned to the background, precluding the identification of relevant binding sites, an issue that likely affects many other datasets. This could explain the lack of general improvement in motif specificity when using narrowPeaks.

To evaluate whether the varying effect of SMInput on recall could be caused by the background model used in PEKA, we selected ten eCLIP datasets with the highest difference in recall observed between the two sets of peaks and analyzed them with STREME [29], which uses shuffled peak sequences as background, thus providing an independent approach to investigate the effects of using SMInput for analysis of eCLIP data. We provided STREME either with sequences from Clippy peaks or narrowPeaks, and then compared the ranking of significantly enriched k-mers retrieved by PEKA or STREME (motifs with *p* < 0.05) in the corresponding in vitro data. In the cases of RBPs where PEKA analysis of narrowPeaks gave better ranking than Clippy peaks, STREME analysis also achieved better ranking with narrowPeaks for all datasets (Additional file 1: Fig. S4c). Furthermore, in the cases of RBPs where PEKA analysis of Clippy peaks gave better ranking than narrowPeaks, STREME analysis also recovered motifs with either higher or similar ranking for Clippy peaks compared to narrowPeaks (Additional file 1: Fig. S4d). This indicates that the lack of general motif improvement from narrowPeaks as compared to Clippy peaks in PEKA is reliable, since the alternative motif analysis approach follows the same trends. We also found that the median number of tXn that overlap with narrowPeaks is around 10-fold lower compared to the Clippy peaks used with PEKA (Fig. 3c). Thus, in spite of using SMInput filtering, we do not find the use of narrowPeaks to improve the general specificity of identified motifs; however, it greatly reduces the sensitivity of the analysis.

Taken together, this analysis demonstrates that the value of SMInput-based correction depends on the specificity of the studied RBP, for example, it can be beneficial for proteins binding to A-rich motifs. We speculate that this might be because RBPs binding to A-rich motifs are minor contributors to the SMInput data, which is rather dominated by proteins binding U-rich motifs. As a result, for proteins such as hnRNPC SMInput likely represents a mixture of foreground and background signal, leading to depletion of the foreground signal from narrowPeaks. Taken together, this analysis demonstrates that it is appropriate for PEKA to perform motif analysis by relying on the intrinsic background modelled from each individual CLIP dataset, without the need to use SMInput filtering.

### Insights into the technical biases of CLIP

**Fig. 4:**
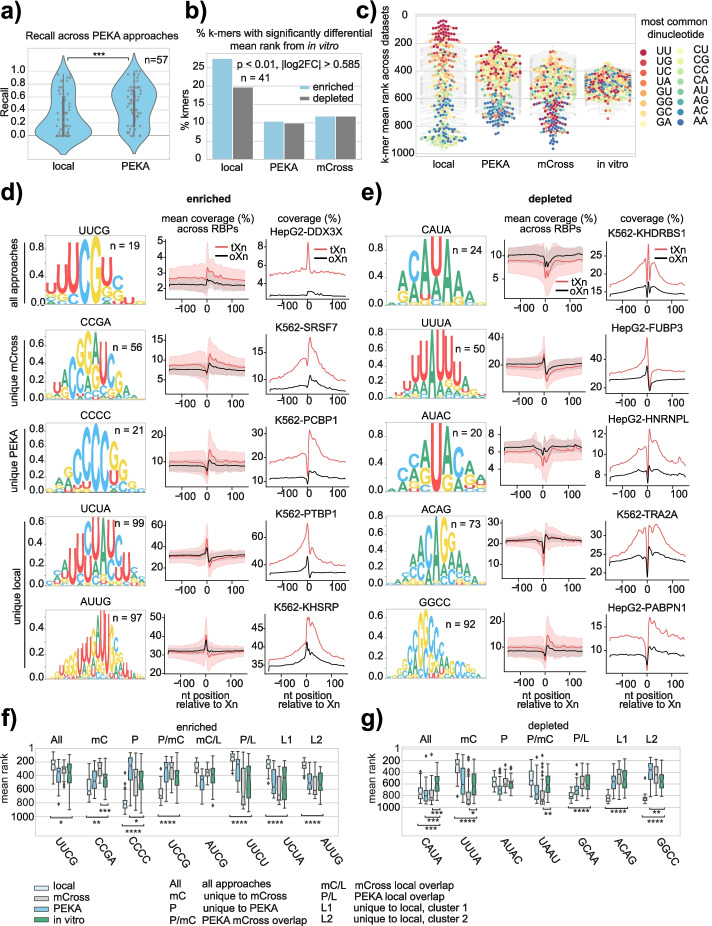
Differential enrichment of motif groups in eCLIP relative to in vitro methods. **a** Recall values for PEKA and “local” approach across 57 eCLIP datasets with available in vitro data (Additional file 8: Table S7). **b** Percentage of k-mers with significantly differential ranking (Welch’s *t*-test *p* < 0.01 and a fold change greater than 1.5 or less than 0.66 between each eCLIP analysis approach and in vitro data. Differential ranking was assessed on the 41 eCLIP datasets for which both mCross (raw scores) and in vitro data were available. **c** Swarmplot shows mean k-mer ranks across RBPs (*n* = 41) in each approach. K-mers which contain two or more of the same dinucleotide are colored by their most common dinucleotide and other k-mers are shown in gray. K-mer ranks in the mCross approach were assigned based on raw scores. **d**,**e** An overview of the differentially enriched (**d**) and depleted (**e**) 5-mers (see “Methods”) in eCLIP analysis approaches relative to in vitro data. In each panel, k-mer groups are arranged from top to bottom as follows: k-mers that are differentially ranked in all approaches (“all approaches”), only in raw mCross (“unique mCross”), only in PEKA (“unique PEKA”) or only in “local” approach (“unique local,” where k-mers were split into 2 clusters based on their sequence). K-mer logo is shown for each k-mer group, with nucleotides scaled proportionally to their frequency at each position in the alignment. The left line plot shows the distribution of mean motif group coverage around tXn (red) or oXn (black) within the “protein-coding gene” region across the 57 eCLIP datasets that have orthogonal in vitro data. Area around the curve shows the standard deviation. The right line plot shows the distribution of motif group coverage around tXn (red) or oXn (black) within the “protein-coding gene” region for a representative RBP that ranked highly in both eCLIP and in vitro datasets for the corresponding motif group. **f**, **g** Boxplots showing distribution of mean motif ranks across RBPs in methods used to analyze eCLIP and in vitro data for the enriched (**f**) and depleted (**g**) motif groups (shown in panels **d**, **e** and in Additional file 1: Fig. S5a,b). For each motif group, we compared the distribution of mean ranks in CLIP analysis approaches with in vitro data to assess the significance of difference with Welch’s *t*-test and the comparisons are marked when significant

To illustrate how PEKA controls for technical biases, we compared the motif ranking between PEKA, mCross, and a simple “local” approach that only examines the local relative motif occurrence in a narrow (−3...3nt) window around tXn sites, normalized by the average occurrence within distal windows (−150…−100 and 100…150nt around tXn), rather than the intrinsic background (see “Methods”). We first analyzed recall across 57 eCLIP datasets for PEKA and the “local” approach (Fig. 4a) to find that PEKA significantly improved recall as compared to the “local” approach. While both PEKA and the “local” approach decrease the biases of regional genomic sequences, the normalization with the intrinsic background used by PEKA can also correct for technical biases at crosslink sites such as those resulting from crosslink and ligation biases, which likely explains its higher agreement with the in vitro data.

To gain insights into the reasons why certain motifs are more or less recoverable from eCLIP than in vitro data, we next compared motif enrichments obtained by the “local” approach, PEKA, mCross or in vitro across a subset of 41 eCLIP datasets (representing 28 distinct RBPs, Additional file 2: Table S1) that had both in vitro and mCross data available. We identified groups of k-mers that were differentially ranked in each method relative to in vitro data (confidence interval > 95% and a fold-change greater than 1.5 or less than 0.66) (Fig. 4a, “Methods”). Interestingly, the “local” approach produced most motifs that were differentially enriched in eCLIP vs in vitro (~27%), and PEKA produced the least (~10%) (Fig. 4b, Additional file 1: Fig. S5a,b).

To investigate the general sequence characteristics of differential k-mers, we visualized the mean rank of each k-mer across datasets for each approach and colored the k-mers according to their most common dinucleotide (Fig. 4c). As expected, the “local” approach was strongly enriched for k-mers in which the predominant dinucleotide is UU. The ranking of such motifs was strongly decreased by PEKA, while they still tend to be among highest ranking motifs, whereas such motifs tend to be the lowest ranking in mCross. Conversely, the “local” approach was strongly depleted for CU, CG, and CC dinucleotides, all of which are efficiently corrected by PEKA and mCross where they rank at levels comparable to in vitro*.* Interestingly, AA-rich motifs are depleted in all CLIP analysis approaches as compared to the in vitro data, with the strongest depletion seen in mCross. These k-mer sequence imbalances in eCLIP data demonstrate the importance of assessing global trends of enriched motifs across large numbers of RBPs to understand both the biases of experimental and computational approaches, as well as the potentially true differences in the binding preferences of RBPs between in vitro and in vivo conditions.

To investigate if the same biases are observed also in other CLIP variants, we expanded our analysis to 19 PAR-CLIP datasets for which in vitro data was available. In PAR-CLIP, crosslinking is performed at a higher wavelength and with the addition of a photoactivatable nucleotide 4-SU, which, upon sequencing, allows for the precise locations of crosslink sites to be identified, based on observing T-to-C transitions. We first performed a recall analysis and observed slightly improved recall in PEKA compared to the “local” approach; however, the change was not significant. Furthermore, the recall of PEKA motifs obtained from PAR-CLIP data was generally lower compared to the RBP-matched eCLIP datasets (Additional file 1: Fig. S5d). We then analyzed the proportion of k-mers that exhibit significant differences in ranking between CLIP and in vitro data to again find that PEKA greatly reduces the number of differential k-mers as compared to the local approach (Additional file 1: Fig. S5e). When we analyzed k-mer ranking with respect to their sequence composition, we observed relatively similar trends as in eCLIP, with U-rich k-mers being highly enriched and C-rich k-mers depleted in the local approach, while the effect is diminished in PEKA k-mers. Interestingly, the bias against AA k-mers that was observed in eCLIP data is not apparent in PAR-CLIP, and instead some bias against GG-containing k-mers is seen in PAR-CLIP (Additional file 1: Fig. S5f).

19 k-mers were differentially enriched in eCLIP by all three analytic approaches (the UUCG group, Fig. 4d) and 24 were differentially depleted (CAUA group, Fig. 4e, Additional file 1: Fig. S5b). Interestingly, these motifs are evenly enriched up to 150nt around tXn sites as compared to oXn sites (Fig. 4d). One of the proteins with the highest ranking of these k-mers (in eCLIP) is the DEAD-box helicase DDX3X, which is a major regulator of cellular RNA condensates and itself contains intrinsically disordered domains (IDRs) with strong condensation propensity [30]. A study reported that DDX3X binds in the vicinity of a motif composed in large part of CG and CGU subsequences, similar to the k-mers found in the UCG-group [31]. It could be speculated that such patterns are better detected by CLIP than in vitro binding data due to their need to assemble “binding-region condensates” on long RNA regions as was observed for TDP-43 [21].

Both PEKA and mCross use a strategy to control for the technical biases of CLIP, and it is thus reassuring that their differential motifs are more similar to each other than to the “local” approach (Fig. 4d,e, Additional file 1: Fig. S5a). To further understand the unique features of each approach, we examined the motifs that were enriched or depleted uniquely in each approach (Fig. 4d,e), or in two approaches (Additional file 1: Fig. S5a,b). This showed that differential motifs identified either by mCross and PEKA tend to be broadly enriched or depleted at over 100-nt region around crosslink sites, whereas as expected, those identified in the “local approach” are enriched or depleted directly at crosslink sites (Fig. 4d,e). We also visualized the extent of the differences in ranks for each motif group, which shows that the greatest drop is seen for the CAUA group for all CLIP analysis approaches as compared to in vitro data, and for the CCCC group for the “local approach” to CLIP analysis (Fig. 4f, d).

Importantly, for all motif groups, we show that PEKA is able to identify such motifs as top-ranking for the RBPs that show strong enrichment (Fig. 4d, e). For example, even for the CAUA group motifs that are found depleted in eCLIP by all approaches as compared to in vitro data, KHDRBS1 is correctly identified as a strong binder of the motif, with a broad enrichment pattern seen around crosslink sites (Fig. 4e). Moreover, PEKA can identify enriched motifs for hnRNPL, TRA2A, and PABPN1 even though they are depleted directly at crosslink sites (Fig. 4e). Thus, in spite of generic imbalances observed in the motifs enriched across all CLIP datasets and a great variety of their positional enrichment patterns, PEKA is able to identify all types of motifs when they mediate high-affinity RBP binding.

### Many eCLIP datasets share similar enriched motifs

**Fig. 5:**
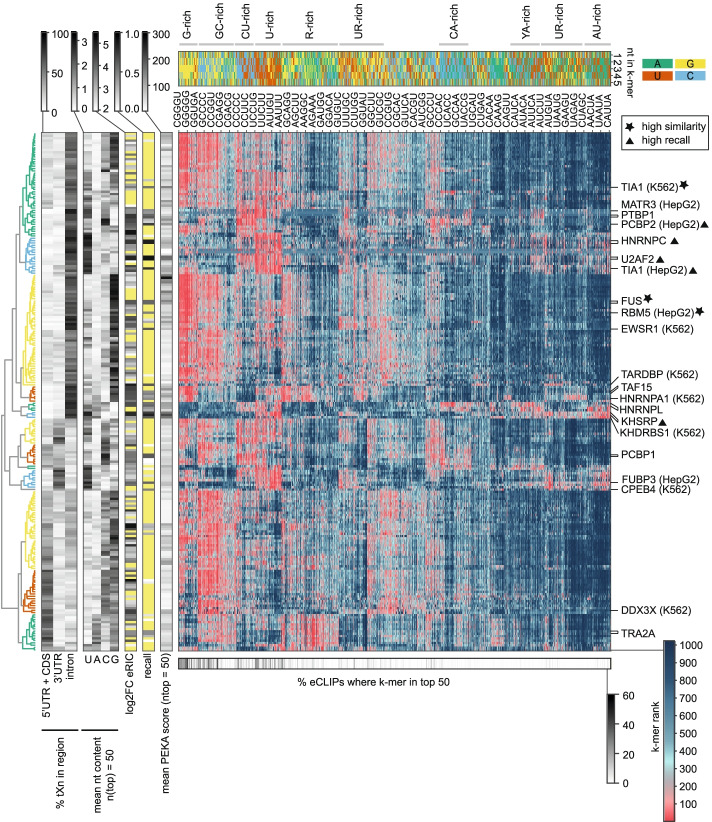
Heatmap of all 5-mers for all available eCLIP datasets. eCLIP datasets are hierarchically clustered by their rank order of 5-mers and regional distribution of high-confidence crosslink sites (tXn) to visualize binding preferences across groups of proteins. Fourteen primary clusters were identified, which are color coded in the dendrogram on the left. K-mers were clustered as described in “Methods.” Additional heatmaps on the left represent (from left to right) (1) regional distribution of thresholded crosslink sites, (2) mean nucleotide composition across top 50 identified k-mers for each dataset, (3) enrichment of the protein in the RNA interactome capture (eRIC [33]), (4) the overlap with orthogonal in vitro methods (i.e., recall), and (5) mean PEKA score across top 50 identified k-mers. RBPs marked on the main heatmap have a recall value > 0.5. A triangle next to the RBP name represents high recall (> 0.8) and a star represents high similarity score (> 0.3). Additional heatmap above the main heatmap represents the nucleotide sequence of each 5-mer and a grayscale heatmap below the main heatmap shows a percentage of eCLIPs, where k-mer ranked among the top 50. Every 20-th 5-mer is labelled on the main heatmap. Yellow fields in grayscale heatmaps indicate missing values

To understand how specific the motifs enriched in each eCLIP dataset are, we performed a systematic cross-motif and cross-RBP comparison of the whole set of eCLIP datasets available from ENCODE [4, 5]. We clustered the data based on the regional crosslinking profiles within protein-coding genes (CDS/UTR/intron) and by the ranks of 5-mers (see “Methods”), which identified groups of eCLIPs with similar motif enrichment signatures (Fig. 5). The first visually apparent feature of this analysis is that regional crosslinking preferences are accompanied by trends towards certain motif preferences. For instance, if crosslinks are primarily in 5′-UTR and CDS, the largest cluster of data shows enrichment in GC-rich motifs. In case of datasets with primary crosslinking in introns, motif enrichment is dominated by three clusters, two large clusters of datasets dominated by G-rich motifs, and a smaller cluster dominated by U-rich motifs. Datasets that crosslink primarily to 3′-UTRs also show enrichment of U-rich or UA-rich motifs (see the blue and yellow cluster, respectively). These clusters likely include binders of the AU-rich elements (AREs) that are common regulators of RNA stability in 3′-UTRs [32]. Moreover, both CDS and intronic datasets are often enriched in C/G-rich motifs. This analysis demonstrates that the common motif preferences in eCLIP data are closely linked to the regional crosslinking profiles within protein-coding genes.

As expected, eCLIP datasets within the largest clusters share highly similar motif preferences and regional profiles. Interestingly, we noticed that RBPs falling within these large clusters generally lack orthogonal in vitro binding data (indicated by the absence of recall) and are often poorly detected in the mRNA interactome proteomics (enhanced RNA interactome capture, i.e., eRIC) [33], and their top 50 ranked k-mers are often G-, U- or GC-rich. It has been reported previously that such k-mers tend to be overrepresented in eCLIP compared to RBNS [3], but the scale of their presence across eCLIP data was not yet examined. Strikingly, several G-rich k-mers were enriched among the top 50 k-mers in more than 50% of all eCLIP datasets.

### Specificity of eCLIP datasets relates to RBP domain types and compositional biases

**Fig. 6:**
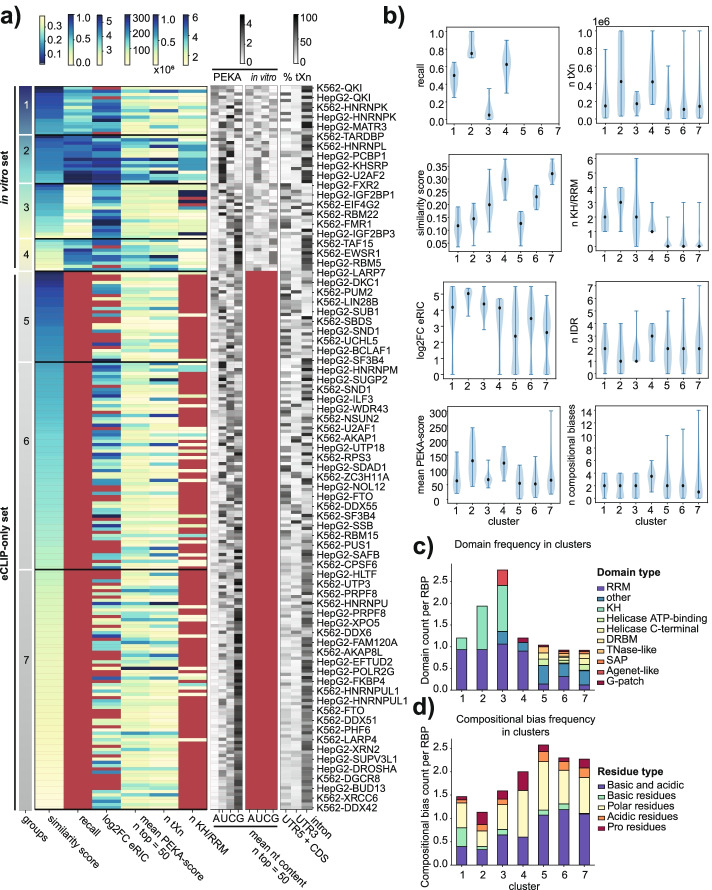
Expected specificity of eCLIP datasets with regard to various features. **a** The left heatmap displays all eCLIP datasets, clustered based on their similarity scores and recall into 7 clusters. For each dataset, the heatmap also shows its enrichment in the mRNA interactome proteomics (log2FC eRIC), the number of KH or RRM in RBP, the average PEKA score across the top 50 ranked k-mers, and the number of tXn in “protein-coding” region (n tXn). Values shown on this heatmap are available in Additional file 7: Table S6. Grayscale heatmaps from left to right show the mean nucleotide content across the top 50 ranked 5-mers for each dataset in PEKA (left) and in vitro data (middle) and % of thresholded crosslinks derived from each transcript region (right). **b** Violin plots show quantitative distribution of features within clusters. In addition to features presented in the heatmap (recall, similarity score, log2FC eRIC, mean PEKA score across top 50 k-mers, number of tXn, and number of KH or RRM domains), they also show the number of intrinsically disordered domains (IDRs) and the total number of compositional biases for RBPs within each cluster. **c, d** Stacked bar plots showing the frequency of a particular domain (**c**) and compositional biases (**d**) per RBP within each cluster. Frequency is expressed as a total count of domain or compositional bias per RBP. Detailed structural information for the analyzed RBPs is available in Additional file 10: Table S9

To further understand how data similarity relates to the various features of RBPs, we divided the eCLIP datasets according to whether or not they had available in vitro data, and then clustered each group based either on a combination of inter-data similarity (similarity score, see “Methods”) and recall, or just on inter-data similarity where no in vitro data was available, obtaining 7 groups of data (Fig. 6a, “Methods”). Most apparent differences are seen between the group of proteins for which in vitro data are available (groups 1–4, i.e., “in vitro set”) and the group for which no data are available (groups 5–7, i.e., “eCLIP-only set”). The in vitro set tends to have high eRIC values, high number of thresholded crosslinks (*n* tXn), and high mean PEKA scores across the top 50 ranked k-mers, whereas the eCLIP-only set tends to have low eRIC values, lower number of tXn, and low mean PEKA scores, indicating that the proteins in the eCLIP-only set do not crosslink well and have low motif enrichment, respectively. The great majority of proteins in the in vitro set contain a K-homology (KH) domain or RNA recognition motif (RRM) and a low-complexity sequence (Fig. 6b), which are the common characteristics of RBPs [34]. Conversely, proteins in the eCLIP-only set rarely contain KH or RRM domain. Higher prevalence of canonical RNA-binding domains in the in vitro set is not surprising, as the great majority of RBNS and RNAC data are in proteins which contain such domains [12, 35]. Interestingly, the in vitro set contains only a small group 4 with a high similarity score, while the eCLIP-only set contains a large group 7 with a high similarity score, accounting for a third (33%) of all eCLIP experiments. Taken together, it is clear that proteins lacking orthogonal in vitro data generally have different features from the rest, and their eCLIP data tends to have lower inter-data specificity (high similarity index) and motif enrichment (low mean PEKA score, eCLIP-only set). This indicates that cross-validation of eCLIP with in vitro data cannot be extrapolated to warrant the specificity of eCLIP data without available in vitro data, which must be taken into account when performing meta-analyses on the whole set of eCLIP data.

The most reliable eCLIP experiments are expected to be in group 2, which includes ~5% of datasets with unique k-mer signatures and high agreement with corresponding RBNS or RNAC data, as indicated by their low similarity index and high recall, respectively. This group of RBPs generally ranked highest in the eRIC experiments, indicating that they crosslink efficiently with RNA, which is consistent with the high number of thresholded crosslinks identified by PEKA. These RBPs contain a median of 3 RRM or KH domains (Fig. 6b). Thus, the canonical RBPs that crosslink well and contain many RNA-binding domains tend to yield specific and reliable eCLIP datasets. In addition to the high cross-validation, RBPs in group 2 have the highest mean PEKA scores across the top 50 ranked k-mers, implying that the coverage of top k-mers around tXn is much higher than around oXn. In other words, binding affinity of these RBPs is strongly sequence-dependent, requiring the presence of one or more high-affinity binding motifs.

The least reliable eCLIP experiments are expected within group 7, containing ~33% of eCLIP datasets with high inter-data similarity, which lack orthogonal in vitro data (Fig. 6a). ~39% of datasets in group 7 are undetected in eRIC experiments, which indicates that they crosslink poorly or do not crosslink at all to RNA. These proteins lack annotated features of RBPs, such as KH or RRM domains (Fig. 6a,b). This increases the likelihood of the signal being dominated by the most common contaminants of eCLIP experiments, which are likely the abundant and well-crosslinking RBPs. Low-specificity datasets in group 7 predominantly enrich for G-rich motifs, with most crosslinking sites originating from introns (Fig. 6a). We note that the same features are also prevalent in group 4, which contains eCLIP datasets that have reasonable agreement with in vitro data, indicating that many of these RBPs directly interact with the G-rich motifs. Nevertheless, these experiments have high inter-data similarity due to the fact that very similar motifs are enriched in groups 4 and 7. However, RBPs in group 4 generally have much higher mean PEKA scores than those in group 7, and thus even though both show enrichment of similar motifs, the extent of enrichment is stronger in group 4 (Fig. 6b).

It is interesting to find many groups with strong regional binding preferences, even though regional preferences had no direct role in clustering the groups (Fig. 6a). For example, groups 1, 2, 4, and 7 all contain predominantly intronic binding. However, G-rich motifs dominate groups 4 and 7, whereas groups 1 and 2 mainly show enrichment of A-rich, C-rich, or U-rich motifs, likely due to its higher data specificity. It is notable that proteins in group 2 contain a median of 3 KH or RRM domains, whereas those in group 4 contain only a median of 1 domain, and do not ever contain a KH domain. Moreover, group 5 commonly shows predominant binding in CDS and 5′-UTR, which tends to be associated with a higher proportion of A-rich motifs. Since A-rich motifs are otherwise rare in eCLIP experiments, their enrichment contributes to the low similarity index of datasets in group 5. We propose two possible explanations why datasets with similar specificity tend to have similar regional binding. First, the signature might reflect similar contaminants; for example, RBPs that bind G-rich motifs in introns might be the common contaminants of datasets from group 7. Second, the link between RBP specificity and regional binding could reflect regional sequence biases.

In addition to the aforementioned differences in the content of RRM/KH domains, we found that our clusters of proteins also differed in other domains. The proteins in the in vitro set very rarely contain domains other than RRM/KH, whereas the proteins in the eCLIP-only set frequently contain helicase domains, TNase-like domain, Tudor domain, and dsRNA-binding domains (DRBM) (Fig. 6c). These domains are less sequence specific, which likely contributes to the generally low eRIC scores of these proteins, and the high similarity scores and low PEKA scores of their eCLIP data.

We also observed differences in the number and types of compositional biases between groups (Fig. 6d). In the in vitro set, group 4 stands out as its proteins generally have higher numbers of intrinsically disordered domains (IDRs) compared to other groups. Interestingly, group 4 generally binds G-rich motifs and has decent recall and high similarity score. Thus, these proteins generally produce specific data, but their IDRs might be prone to formation of condensates or aggregates that can co-purify in immunoprecipitations of other proteins, which could explain high similarity of their data with many other proteins. Finally, we observed differences in the median PEKA score of each group, which is highest in groups 2 and 4, which have the highest recall. Thus, the extent of motif enrichment (as quantified by PEKA score) is related to the extent of cross-validation with in vitro data (Fig. 6a,b).

To understand the generality of insights obtained from eCLIP meta-analysis, we performed the same systematic analysis for the large multi-protein PAR-CLIP resource (Additional file 1: Fig. S6). Unlike the eCLIP analyses, we observed no correlation between unique motif enrichments (low similarity index) and high numbers of RRM/KH domains, and instead we observed a slightly reverse trend in PAR-CLIP, with the most unique data obtained for RBPs that lack or have few KH/RRM domains. Since the purification procedure in PAR-CLIP employs stringent Flag-tag immunoprecipitation and quality control on SDS-PAGE, it is expected that the extent of contaminating signal is lower in PAR-CLIP, and thereby indicates that the high similarity (and common dominance of G-rich motifs) in eCLIP data for RBPs that lack KH/RRM domains is unlikely just a signature of their lower binding specificities. Thus, further studies will be needed to distinguish datasets where high similarity of enriched motifs reflects biological relationships between studied RBPs from those where it reflects common contaminating RBPs or other technical biases in the data.

## Discussion

Here we present PEKA, a user-friendly software that can examine individual CLIP data to reliably discover the motifs that reflect the specificity of purified RBPs. By performing comparative analyses of motif enrichments across 223 eCLIP datasets, we find that the inter-data specificity of enriched motifs, and the extent of motif enrichment (PEKA score), are valuable measures of the RNA sequence specificity of the studied proteins, as well as of data specificity. We also find that the similarity score is closely linked to the number of RRM and KH domains as well as the number of IDRs. Moreover, we demonstrate the value of assessing the positional patterns of enrichment around crosslink sites and show that PEKA can discover motifs with various patterns of enrichment. The motifs that are more enriched in eCLIP data by PEKA and/or mCross as compared to in vitro data tend to have broad enrichment patterns around crosslink sites. Such patterns are reminiscent of the “binding-region condensates” observed on long RNA regions for the condensation-sensitive binding of TDP-43 [29], and it is likely that such condensation-sensitive binding is better detected by CLIP as compared to in vitro data. Moreover, sequence biases of bound genomic regions (caused for example by repetitive elements), effects of RNA modifications, post-translational modifications of RBPs, and multi-protein assembly characteristics that would not be captured by current in vitro data likely contribute to divergence between in vivo and in vitro specificity. Comparisons across further in vivo and in vitro datasets will be valuable to better understand the reasons for the divergence.

It is expected that PEKA score and the resulting top motifs will be influenced by a peak file of choice. Surprisingly, however, we did not observe a general increase in the specificity of identified motifs when using narrowPeaks (Fig. 3a), which are meant to decrease the extent of extrinsic background via the SMInput-based filtering. We found that this approach greatly decreases data sensitivity (Fig. 3c) and can remove foreground signal from eCLIP data (Additional file 1: Fig. S4b). Moreover, we show that the k-mers identified by PEKA correlate well with the mCross k-mers that used a different peak calling approach (Additional file 1: Fig. S3a). Nevertheless, PEKA presents several advantages as compared to mCross. (1) It is able to analyze a larger set of RBPs, since it obtains k-mers with generally good recall even for the 16 RBPs that were excluded from the mCross study (Additional file 1: Fig. S3b) due to insufficient numbers of significantly enriched k-mers and/or low concordance between replicates. (2) Whereas mCross generates metamotifs (weblogos), the results of PEKA are presented as k-mer clusters, which enables it to visualize the more complex and mechanistically relevant enrichment patterns (Additional file 1: Fig. S2), and is also more compatible with further cross-RBP comparative visualizations of motifs. (3) Unlike mCross, PEKA enables regional analysis and visualization of enriched motifs, allowing the user to distinguish between motifs specific to a particular genomic region for the investigated RBP. (4) PEKA code is available open source via GitHub and Bioconda in an accessible and user-friendly manner with adjustable parameters [15] and is integrated into the iMaps platform for reproducible analysis of CLIP data [17].

The eCLIP experimental protocol omits the visualization of purified protein-RNA complexes, which is normally used for experimental optimization of specificity via visual analysis of expected vs. co-purified RBPs [6], and therefore analysis of data specificity is particularly pertinent to eCLIP. Previous studies evaluated successful IP, library complexity, and the number of reproducible CLIP peaks as a signature of data quality [3]; however, these metrics serve mainly as measures of data sensitivity [36]. As a measure of data specificity, previous studies have reported the agreement of enriched motifs between eCLIP with RBNS [3], but have not used such analyses to compare various quantitative metrics of data specificity across datasets. We find that 5% of datasets contain the highest specificity characteristics of high agreement with orthogonal in vitro data and inter-eCLIP data specificity, whereas ~33% of datasets have low inter-eCLIP specificity, lack orthogonal in vitro data, and in most cases show a predominance of G-rich motifs. Notably, such motifs are found enriched even in some eCLIP (but not iCLIP or PAR-CLIP) datasets from the highest quality datasets, such as TIA1 (Fig. 2). It has been hypothesized that these motifs may be bound by co-purified RBPs [3], and our analyses indicate that this contamination affects mainly eCLIP, but not iCLIP and PAR-CLIP datasets of the same proteins. Interestingly, one earlier study reported enrichment of G-rich motifs in PAR-CLIP background [37]; however, we did not detect these motifs commonly enriched in PAR-CLIP data examined in our study (Additional file 1: Fig. S6). Since we find that enrichment of G-rich motifs is seen in datasets with preferential intronic crosslinking, we speculate that G-rich background might result from contamination of chromatin and associated RBPs. Such contamination tends to emerge when cell extracts are too concentrated and viscous [38], and therefore the conditions of CLIP normally recommend sonication and DNase treatment of a well-diluted extract, and visualization of purified protein-RNA complexes to confirm lack of contaminating signal [39].

We find that RBPs with the highest motif enrichments (high mean PEKA scores) and inter-eCLIP data specificity (low similarity score) in eCLIP tend to have several RRM and KH domains (Fig. 6). While this informs on the extent that RBPs might be sequence-specific [40], it also informs on the specificity of eCLIP datasets. Datasets that agree most highly with in vitro data are expected to be most specific (i.e., group 2 in Fig. 6), and these have the highest PEKA scores and inter-eCLIP data specificity. Interestingly, the same RBPs are also most efficiently identified by RNA-crosslinking purification (eRIC), indicating that they crosslink well. Interestingly, all RBPs in group 2 predominantly bind to introns. Thus, it is at present somewhat unclear whether the major differences in motif specificity (similarity index) and enrichment (PEKA score) across eCLIP datasets reflect differences in the extent of biological sequence specificity of studied RBPs, variable capacity of eCLIP to isolate the studied proteins without co-purified RBPs, regional differences in motif-driven RBP recruitment, or a mixture of these factors.

Our analysis of motif enrichments is focused on protein-coding genes because most eCLIP datasets are for RBPs that do not bind to non-coding RNAs (ncRNAs), and partly because the RBPs that do, primarily bind to a few abundant ncRNAs [3] that are not sufficient for motif derivation. Moreover, abundant ncRNAs are highly structured and modified and involve complex and multi-step RNP assembly mechanisms, and therefore they require integrative analysis of multiple types of data and structural modelling before the functional sequence motifs can be extracted [41, 42]. Nevertheless, a subset of eCLIP data is highly enriched in ncRNAs, and clustering of eCLIP by regional crosslinking profiles that include abundant ncRNAs and repetitive RNA elements does lead to smaller clusters [3] compared to the clusters defined by enriched motifs in our study (Fig. 5). Therefore, it is important to note that the similarity index from our study only represents specificity of motifs enriched in protein-coding genes, rather than specificity of eCLIP data as a whole. In the future, it will be valuable to analyze enriched sequence and structural motifs in combination with the types of bound RNA to understand additional features that contribute to the specificity of CLIP datasets. It is also likely that PEKA will prove valuable beyond CLIP for analyses of enriched motifs at many other types of nucleotide-resolution datasets.

## Conclusions

PEKA software enables discovery of enriched motifs from CLIP data that efficiently corrects the technical biases at crosslink sites by using the out-of-peak crosslink sites as a source of background sequences. PEKA provides visualizations of motif clusters across transcript regions to gain insights into motif sub-classes, their distribution patterns around crosslink sites, and their commonalities across datasets and across RNA regions. We anticipate that assessment of the extent of motif enrichment (PEKA score) and inter-data similarity of enriched motifs (the similarity index) will become increasingly valuable as further CLIP datasets become available. This will help to better understand the specificity of CLIP data, as well as the molecular mechanisms that contribute to the specificity of protein-RNA interactions in cells. We provide a web platform for visualization of these motif enrichments and distributions for motif-centric or RBP-centric data exploration for the entire ENCODE resource [18].

## Methods

### Source data and primary analysis

Many variants of CLIP protocol enable identification of crosslink sites by analysis of crosslink-induced features. Here we follow the approach introduced by the iCLIP method, where cDNA truncations serve to identify crosslink sites, which has been adopted by many other variants, including eCLIP [2]. Due to the short time of excitation, ultraviolet light is considered a zero-distance crosslinker that induces the formation of a covalent bond between the RBP and RNA that are in direct contact at the time of irradiation [43]. While it is in principle possible to derive some crosslink sites also from analysis of mutations in eCLIP reads, we did not use this information as these sites were less effective with regard to the specificity of derived motifs [11] and the proportion of reads with such mutations varies between eCLIP datasets in a way that could introduce further technical variation. Thus, to obtain crosslink sites, eCLIP fastq files were downloaded from the ENCODE consortium (Additional file 2: Table S1) [4, 5] and processed to get positions of cDNA truncations [44]. First, adapters were removed with Cutadapt (v3.4) using two rounds of adapter removal to account for double ligations as per the ENCODE standard operating procedure and the unique molecular identifier positioned at the end of the fastq header. We aligned the second read as this contained information as to the crosslink position [45]. We used the nf-core/clipseq pipeline [46, 47] to process the reads, first filtering out reads that aligned to rRNA or tRNA and then aligning to the human genome (GRCh38 primary assembly, Gencode V29 annotation). PCR duplicates were removed using unique molecular identifiers and the crosslink position identified as the coordinate immediately 5′ to the alignment start. eCLIP narrowPeaks in GRCh38 genome build, combined from both replicates and filtered with corresponding SMInput control, were downloaded as BED files from the ENCODE consortium [4, 5].

For hnRNPC [48], hnRNPL [49], TIA1 [50] and TARDBP [51], bed files with positions of cDNA truncations were obtained by standardized iCLIP read processing employing the iMaps web server [17] mapping to the human GRCh38 genome build. For PAR-CLIP samples (Additional file 3: Table S2) [52], fastq files were downloaded from the SRA database. Reads were stripped of the 3′adapter sequence by Flexbar (v2.5) and collapsed to remove PCR duplicates. Next, reads were sequentially mapped to reference transcripts by Bowtie2 (v2.3.2) in the following order by retaining the unmapped reads from the previous to the next mapping step. We started with human pre-rRNA (GenBank U13369.1), followed by rRNA (GenBank NR_023363.1, NR_003285.2, NR_003287.2, NR_003286.2), snRNA, snoRNA, other ncRNA (all from Ensembl, including RN7SL), tRNA (GtRNADb), mtDNA (GenBank AF347015.1), and finally the human genome (GRCh38, primary assembly). The last genome-mapping step was performed by the STAR aligner (v2.5.3a), and only uniquely mapped reads (MAPQ=255) were retained for further processing. T-C transitions were extracted using the SAMtools mpileup command and row_mpile_coverage_plus_TC.pl script [53]. SRA accession codes to all iCLIP and PAR-CLIP samples used for this study are available in Additional file 3: Table S2.

We assigned crosslink sites by positions of cDNA truncations for eCLIP and iCLIP data (located 1nt upstream of 5′ mapped read positions), and by positions of T-C transitions for PAR-CLIP data. We did not use mutations from eCLIP and iCLIP experiments, because mutations were found to be less consistent across replicates for motif discovery [11], the proportion of mutations can vary greatly across experiments, and the causes of mutations can also derive from sequencing errors or divergence from genomic reference sequence, among other confounding issues. Crosslink files of replicate experiments for each RBPs from each study were then merged, such that if multiple replicates identified the same crosslink site, cDNA counts were summed up (as defined in Additional file 3: Table S2).

All remaining analyses of CLIP data were done using newly developed or modified scripts as described below, written in Python version 3.7.3, using GRCh38.p12 primary assembly genome build and GENCODE V39 annotation. For PEKA, all annotated repeat sequences in the input genome were soft-masked using RepeatMasker (v4.1.0) [54]. The annotation file was filtered to retain only entries with transcript support level 1 or 2, in genes where such transcripts were available (FilterGtf.py on GitHub [55]) and the filtered annotation was used to produce a segmentation file with the *get_segments* function from the iCount tool [56, 57]. Regions that failed to be annotated with iCount segment were added to the segmentation file with feature label “genic_other” (ResolveUnnanotated.py on GitHub [55]). The generated segmentation file *sorted.genic_other.regions.gtf* can be downloaded from iMaps webserver for analysis of CLIP data (execution number 199764049250 [58]).

5-mer *z*-scores for 78 RNA-Bind-n-Seq datasets evaluated in [12], which also contains the ENCODE accession numbers for these RBNS datasets and relevant concentrations (Table S3), were kindly provided in batch form by Dominguez laboratory. These *z*-scores can be derived from 5-mer enrichment scores (*R scores*) available from the ENCODE resource [4, 5] by calculating their mean and standard deviation. For the current study, the *z*-scores were obtained using the concentration of RBP that produced the highest enrichment. RNAcompete 7-mer *z*-scores were obtained from [35] web supplementary data. Raw and normalized mCross 7-mer *z*-scores for all ENCODE eCLIP datasets were provided by Zhang laboratory [11]. 7-mer *z*-scores were converted to 5-mer enrichment scores by calculating the arithmetic mean of all 7-mer scores that contain a given 5-mer. 7-mers which contain a given 5-mer more than once were considered as many times as the number of instances of the contained 5mer. For illustration, when calculating the arithmetic mean of PEKA scores for a 5-mer “UUUUU,” the 7-mer “UUUUUUG” would be considered two times (“[UUUUU]UG,” “U[UUUUU]G”). For the ease of reproducing the findings of our study, we provide the 5-mer *z*-scores for RBNS, RNAC, and mCross datasets in Additional file 4: Table S3 and 5-mer rankings derived from these *z*-scores in Additional file 6: Table S5, but alert the readers that all of these originate from the previous studies referenced above.

Enhanced RNA interactome capture (eRIC) data from Jurkat cells was obtained from (Supplementary Data 1 in [33]). For our analyses, we visualized the log2-fold change (FC) in signal intensity in UV irradiated (UV+) over non-irradiated (UV-) eRIC samples.

### Peak calling

For the purpose of motif analysis, peaks of crosslinking events were determined using Clippy V1.4.1 with default parameters and *--intergenic_peak_threshold* set to 5 to enable intergenic peak calling [19, 59]. Clippy is optimized for fast CLIP peak calling informed by genomic annotations. First, the single-nucleotide crosslink signal is smoothed using a rolling mean of window size 10. Next, to determine the minimum height thresholds for peaks, the genome is split into regions based on annotations and crosslink density. For this specific analysis, genes with more than five mapped cDNAs were split into exons and introns and intergenic regions were generated based on smoothed crosslink density. Peak summits are determined using the find_peaks function from the *scipy* Python library [60] and filtered based on their height, with summits below the regional mean of cDNA signal being removed. Additionally, summits with a prominence below this regional mean are discarded. Broad peak regions are calculated from each summit based on the shape of the surrounding signal curve and filtered to contain at least five cDNAs.

### Positionally enriched k-mer analysis (PEKA)

PEKA is a tool for finding enriched sequence motifs from CLIP data available at GitHub [15]. For this study, we used V0.1.6 (deposited to Zenodo [61]) and focused on analysis of crosslink sites in the protein-coding regions. First, thresholding splits the crosslinks into high-confidence thresholded crosslinks (tXn) and reference background crosslinks (oXn). The thresholded crosslink sites (tXn) are indicative of high-occupancy protein binding, and the reference group (oXn) can be thought of as the “intrinsic background” of CLIP experiments, stemming from more transient RNA binding of the protein [6].

The first step is to use regional thresholding to obtain tXn. Each transcript is considered separately, such that all exons (including CDS, UTRs, and ncRNAs) within a gene are combined into one region, and each intron and intergenic region are treated as their own region. Within each region, a cDNA count threshold is determined at which ≥70% (the default, but this percentile can be modified by the user) of the crosslink sites within the region have a cDNA count equal or below the threshold, e.g., if the region contains 10 crosslinks, nine out of which have a cDNA count of 1 and one has a cDNA count of 2, the threshold for that particular region is set to 1. tXn are then identified that have cDNA count above the threshold, and overlap with the peaks that are provided by the user. On the other hand, oXn are all crosslinks that fall outside of peaks. The impact of cDNA count threshold will vary based on the depth of the analyzed CLIP library. Currently available CLIP datasets are dominated by crosslinks identified by a single cDNA, and setting the cDNA count threshold to 70% generally serves to remove the crosslinks with a score of 1 from tXn, as such crosslinks represent the vast majority of the data. Increasing the threshold above 70% decreases the number of identified tXn, which can be detrimental especially for small samples. Conversely, if the cDNA count threshold is set to 0, then all crosslinks within peaks will be used as tXn, which can be beneficial for very small samples, but otherwise generally leads to decreased specificity of enriched motifs by ignoring the quantitative information available from cDNA counts.

With increasingly deeper sequencing data, we expect that this threshold will become increasingly important in distinguishing between crosslinks with higher cDNA counts representing various levels of binding strength. For highly complex data in which the number of crosslinks is very abundant, we found that for the sake of saving memory and reducing the time of computation, we can randomly sample from the assigned tXn and oXn to obtain 1 million tXn and 3 million oXn positions, which yields results that are comparable to using all tXn and oXn positions in all tested datasets. While this sampling is done by the default, it can be turned off by the user.

By default, PEKA examines motif enrichment in the following transcriptomic regions: introns (from both coding and non-coding genes); 3′-UTRs; other protein-coding exon regions (comprised of coding sequence exons and 5′-UTR), non-coding RNAs (comprised of exons from non-coding RNAs); intergenic regions; protein-coding genes (comprised of full sequence of protein-coding genes); and whole genome. Optionally, PEKA also allows separate analysis of 5′-UTRs and coding sequence exons. PEKA also supports the use of repeat-masked genomes, and provides the options to either fully exclude repeat elements from motif enrichment analysis, to plot the enrichment in repeat-masked genome in uppercase letters, and that within the repeats in lowercase letters, or to analyze motif enrichment exclusively in repeating regions.

Next, foreground and background sequences are extracted around tXn and oXn, respectively (Fig. 1b), and used to conduct enrichment analysis separately in each transcriptomic region. Foreground sequences are genomic regions spanning −150…150nt around tXn and are subdivided into the proximal (−20…20nt around tXn) and distal window (−150…−100 and 100…150 nt around tXn). Background sequences consist only of the proximal window and span −20...20nt around oXn. The values used for proximal and distal windows used in our study are set by default in the code, but can be adjusted by the user. However, we recommend that the selected proximal window is not more than −50...50nt, as we rarely see enrichment of relevant motifs further than 50nt from crosslink sites.

For each k-mer, PEKA scans across the foreground and background sequences and records the presence of a k-mer by assigning count 1 if present or 0 if absent (Fig. 1c); for k-mers of odd lengths, the position that coincides with the middle of the k-mer is assigned the count, and for k-mers of even lengths, the position that corresponds to its length divided by a factor of 2 is assigned the count (i.e., 3rd overlapping nucleotide for a 6mer). For sequences located in each genomic region, mean k-mer counts are calculated at each position to obtain k-mer occurrences around crosslink sites. Afterwards, relative k-mer occurrence is calculated by dividing k-mer occurrence at each position with the mean k-mer occurrence across all positions within distal windows of the foreground sequences. Relative k-mer occurrence is calculated separately for the foreground sequences (to get RtXn) and for 100 samples of randomly selected background sequences, for which the sample size corresponds to the number of foreground sequences (to get 100 RoXn distributions).

PEKA calculates enrichment of each k-mer by analyzing the positions (relative to crosslinks) where the k-mer is present above a background level, i.e., “the relevant sequence positions” (Fig. 1e). These positions are identified by analysis of the relative occurrences, such that each sequence position within the proximal window gets its own “position-threshold” value, calculated from the 100 RoXn distributions that represent background. For each position, the calculated RoXn values of all possible k-mers (100×4^k^ values) are combined into a union and then the “position-threshold” is defined as the value at which a specified percentile of evaluated RoXn values fall below that threshold. By default, the percentile defining the “position-threshold” is set automatically based on k-mer length and the number of foreground sequences, but a specific value can also be passed by the user. For each k-mer, the positions that contain RtXn higher than the “position-threshold” value are marked as relevant. Setting a higher percentile to determine the “position-threshold” will generally result in fewer relevant positions, while setting the percentile to 0 will result in all positions within a proximal window to be considered as relevant.

Finally, PEKA score is calculated by analysis of k-mer values at the relevant sequence positions (Fig. 1f). For each k-mer, a mean RtXn (ARtXn, i.e., approximated occurrence around tXn) and 100 mean RoXns (ARoXns, i.e., approximated occurrence around oXn) are calculated across the relevant positions, and PEKA score is calculated as (ARtXn − mean(ARoXn)) / std(ARoXn), where mean(ARoXn) represents the mean of ARoXn values across all 100 samples, and std(ARoXn) represents the standard deviation of ARoXn values across all 100 samples (Fig. 1f). To summarize, normalization with a distal window is used to define the relevant positions around crosslink sites, but only normalization with intrinsic background is used to calculate the PEKA scores.

The k-mers are then ranked by PEKA score from the most to the least enriched and all results are given in a table. In addition, a *p*-value is given for each k-mer, calculated from a distribution of PEKA scores across the dataset. For this, PEKA scores are first converted to *z*-scores by calculating their mean and standard deviation, and then *p*-values are obtained from the distribution of *z*-scores with *scipy.special.ndtr* function (*scipy* v1.6.2). PEKA scores for all datasets analyzed in the scope of this research are available in Additional file 5: Table S4 and 5-mer rankings derived from these scores are available in Additional file 6: Table S5.

To aid the user in the first stages of analysis, PEKA output visualizations of occurrence profiles based on the top 20 (or as many as defined by the user) k-mers grouped by sequence characteristics and occurrence distributions (Additional file 1: Fig. S1a). Sequence similarity is stored as a matrix of pairwise jaccard indices calculated with the Python *textdistance* library [62]. The similarity of k-mer occurrence distributions is measured with (1) Spearman rank correlation coefficient, (2) occurrence maximal values, and (3) max peak position. Matrices containing sequence-based and distribution-based metrics are combined at varying weights, the resulting matrix is used for clustering, and the optimal clusters are selected for visualization based on the lowest standard deviation of occurrence medians within clusters.

### Recall of PEKA motifs against in vitro data

Recall was calculated as a proportion of top 20 motifs from in vitro dataset (RBNS or RNAC) that are found among the top 50 motifs in the corresponding CLIP dataset. Recall values for all analyzed CLIP datasets with available in vitro data are reported in Additional file 8: Table S7. In addition, we report the proportion of top 20 in vitro k-mers recovered by corresponding CLIP data at different thresholds for the number of top CLIP k-mers, as shown in comparison between mCross and PEKA (Additional file 1: Fig. S3a, Additional file 8: Table S7).

In Figs. 2, 3, 5, and 6 and Additional file 1: Fig. S3, S4 and S5, we compared selected eCLIP data (Additional file 2: Table S1) with RBNS or RNAC. If both in vitro datasets were available for a particular protein, we always prioritized RBNS over RNAC for the calculation of recall as RBNS *z*-scores were readily available for 5-mers, whereas RNAC required transformation from 7-mer to 5-mer scores.

### Sequence-based clustering of k-mer groups

For sequence-based clustering of k-mers, individual motifs are first converted into tokens that reflect their sequence properties. Tokens resulting from a k-mer are all its subsequences, each combined with an end number denoting their cumulative incidence within a k-mer from left to right. For example a k-mer “AGGU” is tokenized into “A1,” “G1,” “G2,” “U1,” “AG1,” “GG1,” “GU1,” “AGG1,” “GGU1,” and “AGGU1.” K-mer subsequences are marked with a number in the order in which they occur in the k-mer sequence. For example, two guanosines in the example k-mer produce two distinct tokens “G1” and “G2,” one for each nucleotide. After tokenization, pairwise Jaccard similarity is calculated for all k-mers in the group. Jaccard similarity is a quotient of the number of shared tokens between two compared motifs and the number of all tokens in the union formed by the k-mers. The k-mers are then clustered with an affinity propagation method, based on the resulting similarity matrix. Affinity propagation clustering was implemented with the *scikit-learn* v0.21 Python library, using the damping parameter of 0.5, the maximum allowed number of iterations set to 1000, and the number of convergent iterations set to 200. Affinity propagation clustering automatically determines the number of resulting clusters.

For Additional file 1: Fig. S2, the Jaccard similarity matrix was obtained for the top 20 k-mers as described and converted to the distance matrix (1-similarity matrix) for K-means clustering (implemented with the *scikit-learn* v0.21 Python library) to split the motifs into two clusters.

### k-mer logos and consensus sequences of PEKA k-mer groups

To visually represent each k-mer group, obtained after sequence-based clustering, we used sequence logo representations. These were created by k-mer multiple-sequence alignment (MSA) transformed to position-frequency matrix (PFM), as follows. First, pairwise sequence alignments of k-mers are obtained by employing global Needleman-Wunsch algorithm with the *skbio.alignment* module [63], version 0.5.1, setting the score for a nucleotide match to 2 and the mismatch score to −1. Other scoring parameters are left on their default settings: penalty for opening the gap is set to 5, the penalty for gap extension is 2, and terminal gaps in alignment are not penalized. Then, pairwise alignments are collated into the MSA, starting with the highest scoring alignment (in case there are multiple alignments with the same score, the one that is the first by alphabetical sorting is taken) and aligning the second best pairwise alignment containing one of the motifs already included in the MSA to it. This process is repeated with the next best scoring pairwise alignment, until all k-mers are aligned in the MSA. The MSA is then transformed into a PFM, which denotes the frequency of each nucleotide at each position within the alignment. PFM is used to plot sequence logos with the *logomaker* (v0.8) module [64]. By using a rolling window of a predefined length, a motif consensus can also be determined from the PFM by sliding the window across all PFM positions and summing the occurrences of nucleotides within a window. Where the sum is greatest, the majority consensus sequence is derived from the PFM. In case of ties between two or more nucleotides, IUPAC nucleotide notation is used. In case of multiple windows with the same highest scoring sum, the first window in the PFM to get that score is used to derive the consensus sequence. It should be noted that k-mer logos are not an accurate representation of the binding motifs, as PFMs are generated solely based on the sequence alignment of k-mers. Thus, k-mer logos do not necessarily reflect the precise relative positioning of the k-mers or their frequency in the foreground sequences that were used to identify the k-mers. Rather, k-mer logos are used to aid in the visualization of the common sequence features of each investigated group of k-mers.

### Clustering eCLIP datasets

For Fig. 5, we clustered eCLIP datasets based on their distribution within the regions comprising protein-coding genes (namely 5′-UTR, CDS, 3′-UTR, and introns), as well as on their 5-mer ranks. We first calculated cosine similarities between datasets for the regional information and 5-mer ranks separately, and then added the similarity matrices with weights 0.3 and 0.7, respectively. Then, we transformed the combined cosine similarities into cosine distances and used these to perform hierarchical clustering with the *scipy.hierarchy* (v1.7.3) module.

For Fig. 6, we first split the eCLIP datasets into two groups based on whether or not they had available orthogonal in vitro data and then performed k-means clustering (implemented with the *scikit-learn* v0.21 Python library) on each group, using either an equally weighted combination of similarity index and recall, or just similarity index where no in vitro data was available. Prior to clustering, we normalized recall and similarity index across eCLIP datasets using min-max scaling, to ensure an equal contribution of these parameters to clustering. eCLIP datasets with available in vitro data were split into 4 clusters and datasets without available in vitro data were split into 3 clusters. For heatmap visualization, we arranged clusters in each group by their median similarity index, and additionally, datasets within each cluster were arranged in ascending order based on their similarity index. eCLIP clusters and data related to the main heatmap in Fig. 6a are available in Additional file 7: Table S6.

### Similarity score

Similarity score compares how similar the top motifs of a particular eCLIP dataset are relative to other datasets. Similarity score was obtained by calculating pairwise overlap ratios on the top 50 k-mers for all eCLIP datasets and then calculating the mean of these overlap values for each dataset. A similarity score of 0 would indicate that the top 50 of k-mers in a certain dataset were not ranked among the top 50 in any other dataset. In contrast, higher values of similarity score indicate that top motifs of a specific dataset overlap with top motifs in many other datasets. Similarity scores for all eCLIP and PAR-CLIP datasets are available in Additional file 7: Table S6 and Additional file 9: Table S8, respectively.

### Metaprofile of average motif coverage around crosslinks

This script plots metaprofiles of average motif coverage and is available on GitHub [65]. It visualizes the average k-mer coverage of a motif group around crosslink sites within specified transcriptomic regions (corresponding to regions described in PEKA). For this study, the analysis is performed on the full set of user-provided crosslink sites, but optionally these sites can be filtered to determine thresholded crosslinks (tXn, as described above).

Sequences flanking the crosslink sites (default window is −150...150 around the crosslink site, but it can be modified by the user) are scanned with a rolling window equal to k-mer length to find parts of the sequence that match k-mers from the investigated motif group. All positions containing a motif from the investigated group are given a score corresponding to the cDNA count of the evaluated crosslink position and the remaining positions are scored 0. Scores at each position around crosslinks in the assessed region are summed and divided by the total cDNA count of all evaluated crosslinks to generate the coverage showing the percent crosslink events overlapping with any k-mer from the group at each position. Optionally, the user can select to visualize the coverage unweighted by cDNA count (the setting used in this study), in which case all positions containing a motif from the investigated group are given a score of 1, remaining positions are scored 0; scores at each position around crosslinks in the assessed region are summed and divided by the number of evaluated crosslink sites to get the coverage.

Finally, coverage distributions are smoothed using rollmean function with window size of 6 (can be modified by the user) and the metaprofiles for the list of analyzed crosslink files, i.e., samples, are plotted on the same graph. For analysis in Fig. 5, we calculated the average motif group coverage weighted by cDNA scores around tXn and oXn in the “whole gene” region for 24 eCLIP datasets.

For the web interface (see [18]), we calculated metaprofiles of average motif coverage across 26 motif groups encompassing all 5-mers, for all eCLIP datasets. For input, we used thresholded crosslink sites in the “whole gene” region, obtained by PEKA, either removing crosslinks from genomic repeats (no repeats) or not (with repeats). We calculated the weighted motif coverage for each motif group in each sample for “intron,” “other exon,” “3′-UTR,” and the “whole gene” region. For each region, we visualized the coverage profiles for 40 datasets with the highest maximal coverage value within the window −50…50 around crosslink sites in heatmap format, clustering the datasets based on the metaprofile similarities and arranging the datasets within each cluster by falling max coverage values.

For the web interface, we also created regional scatterplots for each motif group (see an example at [66]) displaying on the *y*-axis the maximal value of motif group coverage for each dataset in the window −50...50 around crosslink sites for the analyzed region, and on the *x*-axis the maximal enrichment in the selected dataset compared to all datasets at the same position (*z*-score). *Z*-score value was obtained at each position within the window −50...50 around crosslink sites by calculating the difference between the coverage of the investigated dataset and the mean coverage across all datasets at the same position, and dividing the resulting value with a standard deviation of coverage values at specified position. The scatterplots display a maximal *z*-score achieved by each dataset.

### Generation of differentially ranked motif groups between in vitro data and data produced by eCLIP or PAR-CLIP

The differentially enriched motif groups for Fig. 4 were generated as follows. We obtained k-mers that were differentially enriched between in vitro approaches (RBNS or RNAC) and eCLIP motifs identified by PEKA, mCross or the “local” approach (RtXn) for all RBPs where data were available for at least one in vitro methods and for mCross (Additional file 2: Table S1). For each k-mer, we compared its rank distribution across eCLIP datasets with the corresponding in vitro proteins and performed Welch’s *t*-test to obtain a *p*-value. For each approach, we then extracted significantly differential k-mers that had a *p*-value < 0.05 and a fold change greater than 1.5 or less than 0.66. Finally, differential k-mers were grouped based on the overlap between the analysis approaches and whether they were enriched or depleted in eCLIP, relative to in vitro data (Additional file 1: eFig. S5a,b).

To confirm that RBNS and RNAC datasets were indeed comparable enough to be combined into one category, we calculated the Pearson correlation coefficient between the mean k-mer ranks in differential groups of each method on 11 RBPs that were evaluated in both methods and had overlap with eCLIP. The mean ranks showed moderate correlation (Pearson correlation coefficient of 0.54) (Additional file 1: Fig. S5c), and when differences in median ranks between groups were evaluated with Welch’s *t*-test, they were nonsignificant (*p*>0.05).

To select an example RBP for each k-mer group shown in Fig. 4d,e, we considered eCLIP datasets with available in vitro data. For each eCLIP dataset, we calculated its mean k-mer rank for the motif group in PEKA and in the corresponding in vitro data. Then, we calculated the mean value between PEKA and in vitro k-mer ranks and used this to find the RBP which had the highest (or second highest in case of the AUAC group) enrichment of this motif group in both datasets (Fig. 4d,e). In the case of group UUCG, we did not manage to find a dataset among those with available in vitro data that would enrich for these k-mers; therefore, we considered all eCLIP experiments and found DDX3X in both cell lines to be among the top three datasets for this motif group. In addition, a study reported that DDX3X binds in the vicinity of a motif composed of similar k-mers as in UUCG group [35], thus we selected DDX3X in HepG2 cells as an example dataset for this motif group (Fig. 4d).

### Analysis of domain types and compositional biases

We downloaded gff files from UniProt for all analyzed RBPs and filtered the features for domains and compositional bias. If a specific domain or compositional bias occurred less than 5 times across all datasets, we marked it as “other.” This was then presented in Fig. 6 by counting the number of each domain type and compositional bias within each cluster of datasets, and normalizing the counts with the number of RBPs within the cluster to generate stacked bar plots. Number of compositional biases and KH/RRM domains are available for all eCLIP proteins in Additional file 7: Table S6, for all PAR-CLIP proteins in Additional file 9: Table S8, and more detailed structural information for all RBPs presented in this manuscript is available in Additional file 10: Table S9 (related to Fig. 6c,d).

### STREME

We ran STREME (v5.4.1) on sequences extracted from Clippy peaks and narrowPeaks for eCLIP datasets, shown in Additional file 1: Fig. S4c,d. Sequences were encoded in standard RNA alphabet, the width of motifs (*w*) was fixed at 5 nucleotides, for easier comparison with 5-mers, produced by PEKA. The objective function to optimize for motif discovery was set to differential enrichment (default), the number of seeds to evaluate for each width was set to 100, patience was set to 10, and n-order of shuffle was set to 2 (default). STREME stopped after 10 consecutive motifs exceeded the *p*-value threshold (0.05).streme --p fasta_file --w 5 --objfun de --neval 100 --patience 10 --order 2 --rna

To convert STREME motifs to k-mers, we decoded their consensus sequences based on the IUPAC nucleotide code. For example, the motif “UCWUC” was to be converted into two k-mers “UCAUC” and “UCUUC”. Only the k-mer which ranked most highly in the in vitro data was used to represent the STREME motif.

## Supplementary Information

Additional file 1: Supplementary Figures 1-6.

Additional file 2: Table S1 with accession codes and metadata for ENCODE eCLIP datasets used in this study and specifies eCLIP datasets that were used to generate the Figs. 1, 2, 3, 4, 5 and 6 and Additional file 1: Fig. S2, S3, S4 in this manuscript.

Additional file 3: Table S2, which lists all iCLIP and PAR-CLIP experiments used in this manuscript and their accession codes (Fig. 2, Additional file 1: Fig. S5, S6).

Additional file 4: Table S3, which lists 5-mer z-scores for RBNS, RNAC and eCLIP datasets analysed with mCross, which were obtained as described in Methods.

Additional file 5: Table S4, which lists 5-mer PEKA scores for eCLIP datasets processed with Clippy peaks and narrowPeaks, iCLIP and PAR-CLIP datasets.

Additional file 6: Table S5, which lists 5-mer ranks for all datasets used in this study, i.e. eCLIP datasets analysed with PEKA (using Clippy peaks or narrowPeaks), eCLIP datasets analysed with mCross, eCLIP and PAR-CLIP datasets analysed with ‘local’ approach, as well as iCLIP, PAR-CLIP, RBNS and RNAC datasets. For RBNS, RNAC and mCross, additional data are available, which are not shown in the Figures.

Additional file 7: Table S6, which includes data related to Fig. 6 of this manuscript, namely similarity index, recall, number of KH/RRM domains, number of IDRs, number of LCRs, log2FC eRIC, number of thresholded crosslinks in the ‘protein-coding’ region and mean PEKA score across top 50 k-mers for eCLIP datasets. (XLS 60 kb)

Additional file 8: Table S7, which includes recall values for eCLIP datasets analysed with PEKA (using Clippy peaks or narrowPeaks), eCLIP datasets analysed with mCross, as well as iCLIP and PAR-CLIP datasets analysed with PEKA (using Clippy peaks) (related to Figs. 2, 3, 4, 5, 6 and Additional file 1: Fig. S3, S5, S6).

Additional file 9: Table S8, which includes data related to Additional file 1: Fig. S6 of this manuscript, namely similarity index, recall, number of KH/RRM domains, number of IDRs, number of LCRs, log2FC eRIC, number of thresholded crosslinks in the ‘protein-coding’ region and mean PEKA score across top 50 k-mers for PAR-CLIP datasets.

Additional file 10: Table S9, which includes structural data retrieved from Uniprot related to Fig. 6.

Additional file 11. Review history.

## Data Availability

The source code for PEKA software is available at GitHub [15] and is incorporated as part of the iMaps web platform [17], where it can be used for interactive and password-protected analysis of uploaded CLIP data, and for the visualization of meta-analysis across public data [18]. PEKA is licensed under the open-source GNU General Public License v3.0 and the version used for this study (v0.1.6) was deposited to Zenodo (DOI: 10.5281/zenodo.6984815) [61]. Code for processing the ENCODE eCLIP data is available at ulelab/peka-eclip GitHub repository [44] and the nf-core/clipseq pipeline is available at [47]. The source code for plotting metaprofiles of average motif coverage is available at [65]. Fastq files and narrowPeaks for all eCLIP datasets and SMInput controls analyzed in this manuscript are available from the ENCODE consortium [4, 5], with corresponding accession codes and download links listed in Additional file 2: Table S1. PAR-CLIP data analyzed in this manuscript were obtained from various sources and can be downloaded from GEO or the SRA database (Additional file 3: Table S2). iCLIP data analyzed in this study was obtained from GEO or Arrayexpress databases (Additional file 3: Table S2). 5-mer *z*-scores for RNA-Bind-n-Seq datasets were provided by Dominguez laboratory [12], RNAcompete 7-mer *z*-scores were obtained from [35] web supplementary data, and raw and normalized mCross 7-mer *z*-scores were provided by Zhang laboratory [11]. 5-mer *z*-scores derived from original datasets as described in “Methods” are available in (Additional file 4: Table S3), and corresponding 5-mer ranks are available in Additional file 6: Table S5. 5-mer PEKA scores and corresponding ranks for all CLIP datasets represented in this manuscript are available in Additional file 5: Table S4 and Additional file 6: Table S5, respectively. Data related to Fig. 6 and Additional file 1: Fig. S6 of this manuscript, namely similarity index, recall, number of KH/RRM domains, number of LCR, log2FC eRIC, and mean PEKA score across top 50 k-mers, are available in Additional file 7: Table S6 and Additional file 9: Table S8, respectively. Recall values for eCLIP datasets analyzed by PEKA and with raw and normalized mCross, as well as iCLIP and PAR-CLIP data are available in Additional file 8: Table S7. More detailed structural data related to Fig. 6c,d is available in Additional file 10: Table S9. Enhanced RNA interactome capture (eRIC) data from Jurkat cells is available from ([33], Supplementary Data 1).
