# Supplementary material for: Positional motif analysis reveals the extent of specificity of protein-RNA interactions observed by CLIP
Source: Genome Biol. 2022 Sep 9;23:191. doi: 10.1186/s13059-022-02755-2 (PMC9461102; doi:10.1186/s13059-022-02755-2)
Supplement: Supplementary file 1 — Additional file 1: Supplementary Figures 1-6. [file 13059_2022_2755_MOESM1_ESM.pdf]

Fig S1

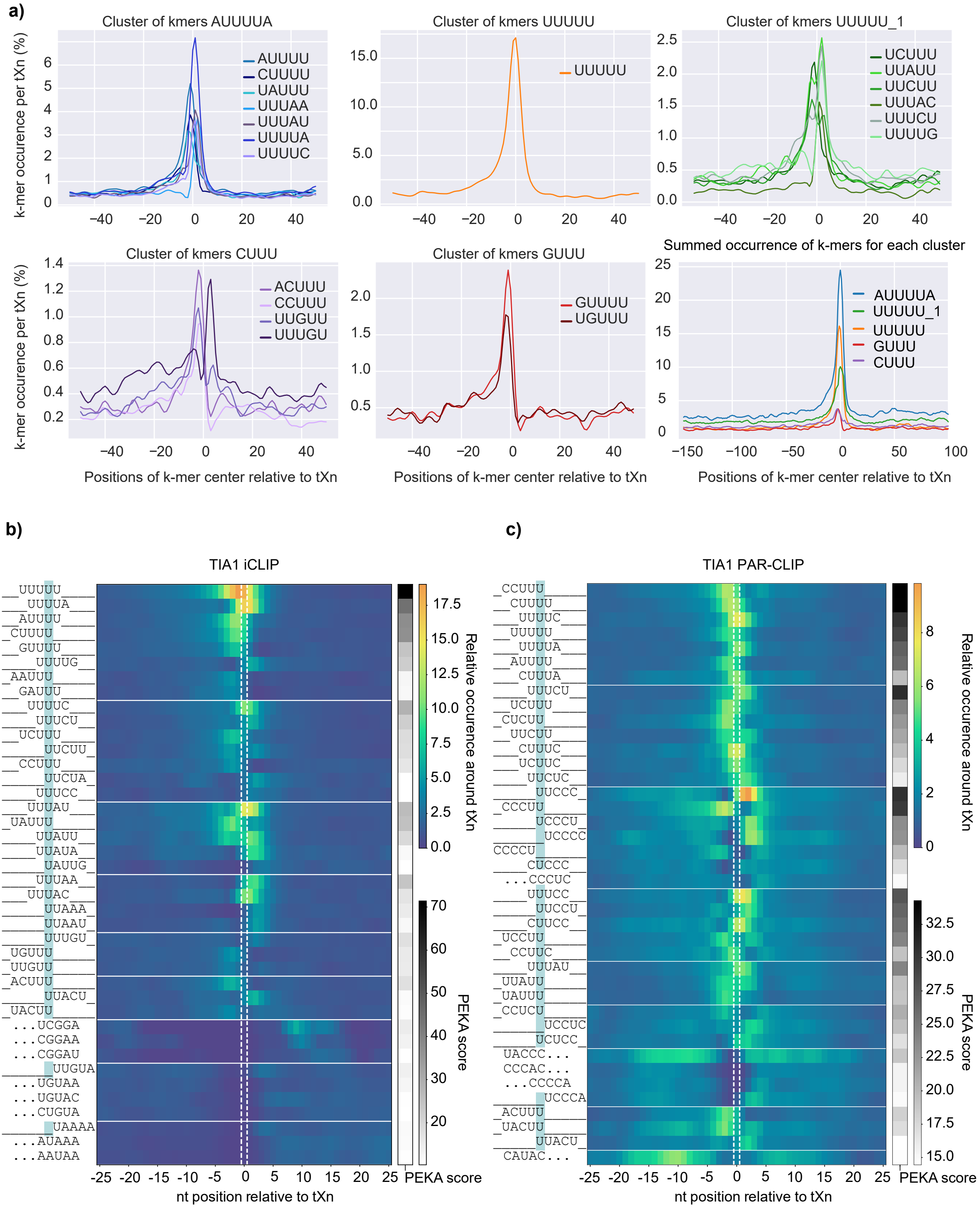
a)
Cluster of kmers AUUUUA
AUUUU
CUUUU
UAUUU
UUUAA
UUUAU
UUUUA
UUUUC
Cluster of kmers UUUUU
UUUUU
Cluster of kmers UUUUU_1
UCUUU
UUAUU
UUCUU
UUUAC
UUUCU
UUUUG
k-mer occurence per tXn (%)
Cluster of kmers CUUU
ACUUU
CCUUU
UUGUU
UUUGU
Cluster of kmers GUUU
GUUUU
UGUUU
Summed occurrence of k-mers for each cluster
AUUUUA
UUUUU_1
UUUUU
GUUU
CUUU
Positions of k-mer center relative to tXn
b)
TIA1 iCLIP
UUUUU
UUUUA
AUUUU
CUUUU
GUUUU
UUUUG
AAUUU
GAUUU
UUUUC
UUUCU
UCUUU
UUCUU
CCUUU
UUCUA
UUUCC
UUUAU
UAUUU
UUAUU
UUAUA
UAUUG
UUUAA
UUUAC
UUAAA
UUAAU
UUUGU
UGUUU
UUGUU
ACUUU
UUACU
UACUU
...UCGGA
...CGGAA
...CGGAU
UUGUA
...UGUAA
...UGUAC
...CUGUA
UAAAA
...AUAAA
...AAUAA
Relative occurence around tXn
PEKA score
nt position relative to tXn
c)
TIA1 PAR-CLIP
CCUUU
CUUUU
UUUUC
UUUUU
UUUUA
AUUUU
CUUUA
UUUCU
UCUUU
CUCUU
UUCUU
CUUUC
UCUUC
UUCUC
UUCCC
CCCUU
UCCCU
UCCCC
CCCCU
CUCCC
...CCCUC
UUUCC
UUCCU
CUUCC
UCCUU
CCUUC
UUUAU
UUAUU
UAUUU
CCUCU
UCCUC
UCUCC
UACCC...
CCCAC...
...CCCCA
UCCCA
ACUUU
UACUU
UUACU
CAUAC...
Relative occurence around tXn
PEKA score
nt position relative to tXn

Fig S1 | Visualisation of PEKA results

a) Positional motif clusters of top 20 ranked motifs for TIA1 iCLIP in HeLa cell line. After ranking k-mers based on their PEKA-score, top *n* k-mers are clustered, based on their sequence and their occurrence distribution around tXn. Smoothed occurrence profiles for k-mers within each cluster are plotted together on a graph and each cluster is represented by its consensus sequence. The final plot shows summed k-mer occurrences for each cluster to elucidate which k-mer groups dominate the RBP's binding landscape and what is the group's approximate distribution pattern. **b,c)** Heatmaps showing relative k-mer occurrences around tXn for 40 most enriched 5-mers for b) TIA1 iCLIP (HeLa cells) and c) TIA1 PAR-CLIP (HEK293 cells). K-mers are clustered based on their sequence and on the left of the heatmaps, their sequences are aligned with the position of relative occurrence maximum. The blue line which spans across labels highlights the most frequently crosslinked nucleotide in the k-mer.

Fig S2

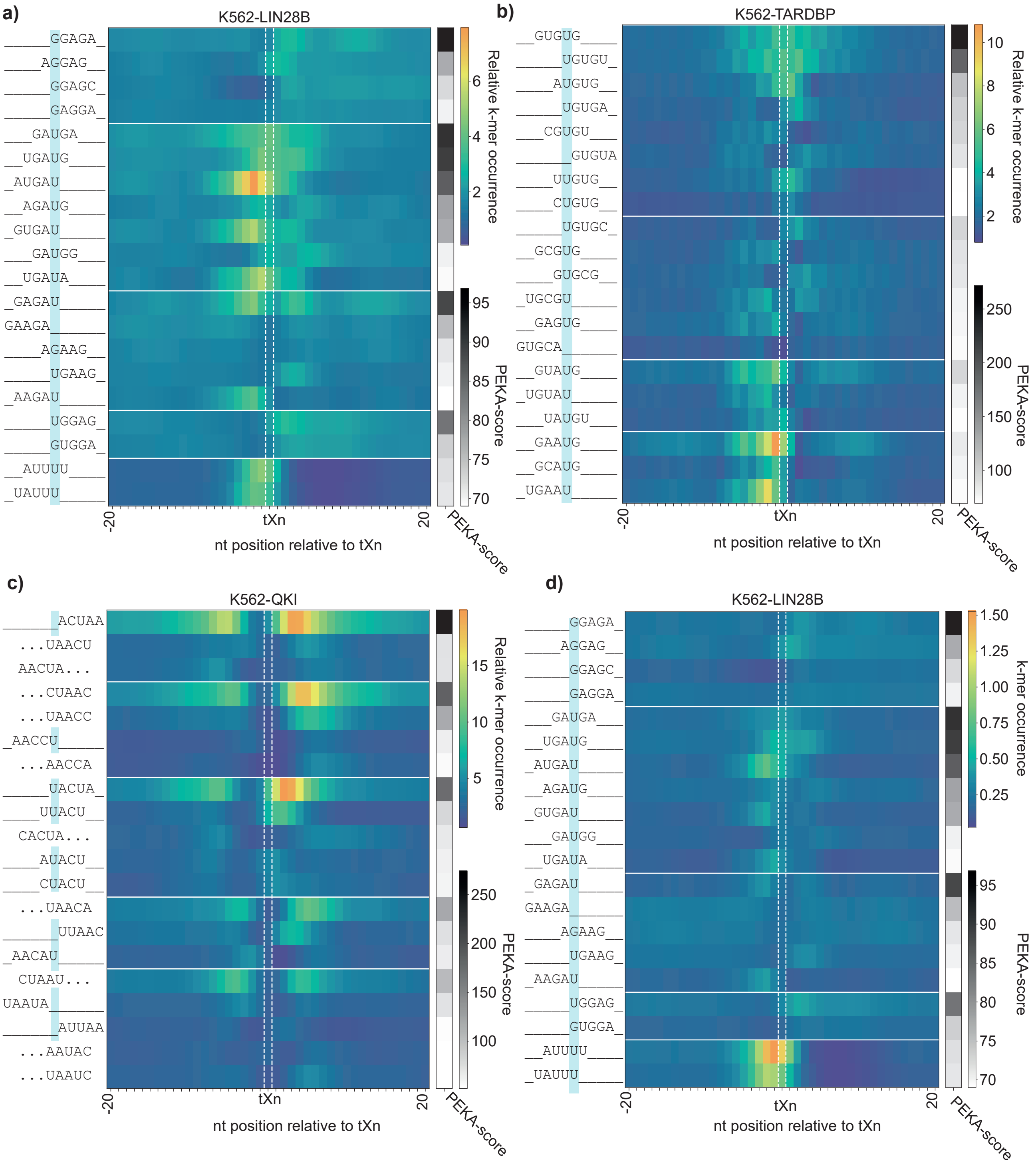
a)
K562-LIN28B
GGAGA
AGGAG
GGAGC
GAGGA
GAUGA
UGAUG
AUGAU
AGAUG
GUGAU
GAUGG
UGAUA
GAGAU
GAAGA
AGAAG
UGAAG
AAGAU
UGGAG
GUGGA
AUUUU
UAUUU
-20
tXn
20
nt position relative to tXn
Relative k-mer occurrence
PEKA-score
PEKA-score
b)
K562-TARDBP
GUGUG
UGUGU
AUGUG
UGUGA
CGUGU
GUGUA
UUGUG
CUGUG
UGUGC
GCGUG
GUGCG
UGCGU
GAGUG
GUGCA
GUAUG
UGUAU
UAUGU
GAAUG
GCAUG
UGAAU
-20
tXn
20
nt position relative to tXn
Relative k-mer occurrence
PEKA-score
PEKA-score
c)
K562-QKI
ACUAA
...UAACU
AACUA...
...CUAAC
...UAACC
AACCU
...AACCA
UACUA
UUACU
CACUA...
AUACU
CUACU
...UAACA
UUAAC
AACAU
CUAAU...
UAAUA
AUUAA
...AAUAC
...UAAUC
-20
tXn
20
nt position relative to tXn
Relative k-mer occurrence
PEKA-score
PEKA-score
d)
K562-LIN28B
GGAGA
AGGAG
GGAGC
GAGGA
GAUGA
UGAUG
AUGAU
AGAUG
GUGAU
GAUGG
UGAUA
GAGAU
GAAGA
AGAAG
UGAAG
AAGAU
UGGAG
GUGGA
AUUUU
UAUUU
-20
tXn
20
nt position relative to tXn
k-mer occurrence
PEKA-score
PEKA-score

Fig S2 | PEKA detects different binding modes of RBPs and motifs with complex patterns

a, b, c) Heatmaps of relative k-mer occurrence around tXn for 20 most enriched 5-mers for **a)** LIN28B eCLIP, **b)** TARDBP eCLIP and **c)** QKI eCLIP in K562 cell line. K-mers are clustered based on their sequence and on the left of the heatmaps, their sequences are aligned with the position of relative occurrence maximum. The blue line which spans across labels highlights the most frequently crosslinked nucleotide in the k-mer. **d)** Heatmap of k-mer occurrence around tXn for 20 most enriched 5-mers for LIN28B eCLIP in K562 cell line. K-mers are clustered in the same manner as described for panels a, b and c.

Fig S3

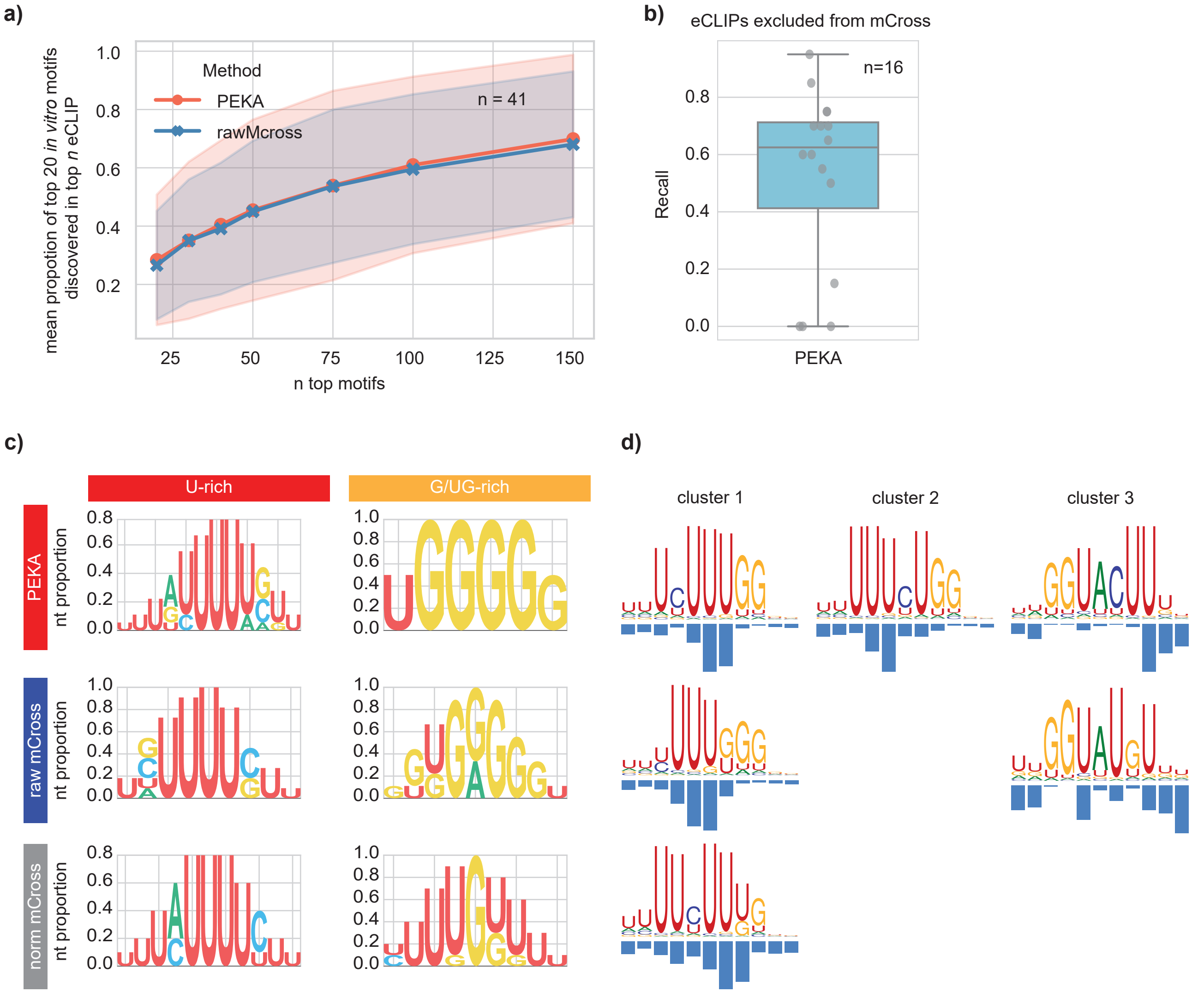

a)
mean propotion of top 20 in vitro motifs discovered in top n eCLIP
Method
PEKA
rawMcross
n = 41
n top motifs
b)
eCLIPs excluded from mCross
n=16
Recall
PEKA
c)
U-rich
G/UG-rich
PEKA
raw mCross
norm mCross
nt proportion
d)
cluster 1
cluster 2
cluster 3

Fig S3 | Comparison of PEKA and mCross motif analysis.

a) Comparison of PEKA and raw mCross in their ability to recover the top 20 motifs from *in vitro* dataset in corresponding eCLIP data. The lines show a mean percentage of top 20 *in vitro* 5-mers that were recovered among the top *n* motifs in each method, across 41 eCLIP datasets for which RBNS or RNAC data was available (representing 28 distinct RBPs in total). The shaded area represents the standard deviation across evaluated datasets at a threshold of *n* top PEKA/mCross k-mers. **b)** Boxplot of recall values for PEKA analysis of eCLIP datasets that did not yield results in mCross (i.e., mCross result is given as 'no motif for these datasets'). **c)** k-mer logos for two clusters of top 20 5-mers (see Methods) obtained for TIA1 in HepG2 cell line by PEKA, raw mCross, and normalised mCross. All three approaches to motif analysis retrieved the canonical U-rich TIA1 motifs. PEKA and raw mCross also found the G-rich motifs enriched among the top 20 5-mers, but these were removed by normalisation process, performed by mCross. **d)** Sequence logos for TIA1 eCLIP in HepG2 cell line downloaded from the mCross base [1]. The sequence logos were derived from the top 10 7-mers in the dataset, ranked by normalised mCross z-scores. 7-mers were clustered to produce sequence logos with Stamp [2] as described in [1].

Fig S4

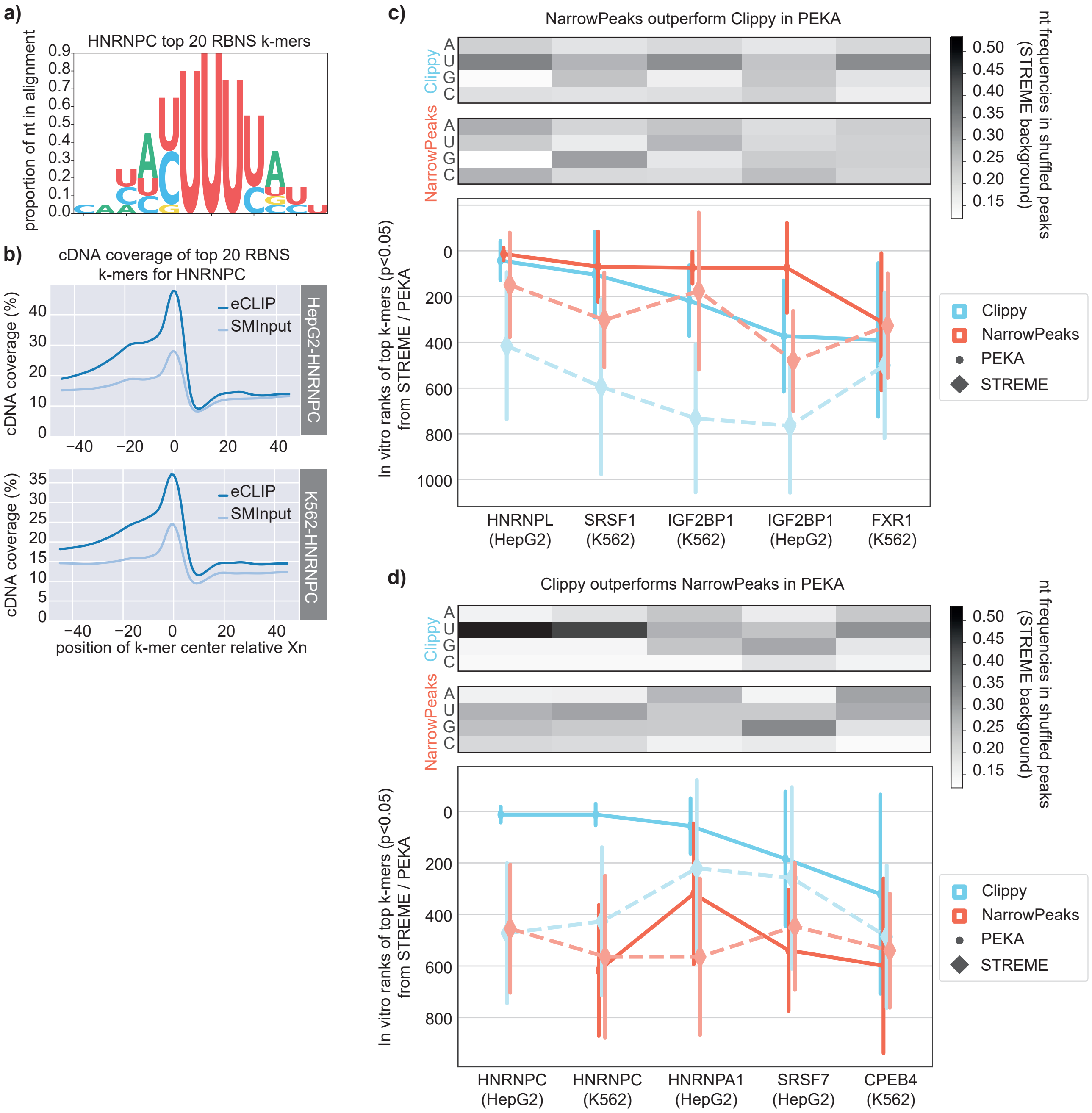

a)
HNRNPC top 20 RBNS k-mers
proportion of nt in alignment
0.0
0.1
0.2
0.3
0.4
0.5
0.6
0.7
0.8
0.9
b)
cDNA coverage of top 20 RBNS k-mers for HNRNPC
cDNA coverage (%)
eCLIP
SMInput
HepG2-HNRNPC
K562-HNRNPC
−40
−20
0
20
40
position of k-mer center relative Xn
c)
NarrowPeaks outperform Clippy in PEKA
Clippy
NarrowPeaks
A
U
G
C
nt frequencies in shuffled peaks (STREME background)
0.15
0.20
0.25
0.30
0.35
0.40
0.45
0.50
In vitro ranks of top k-mers (p<0.05) from STREME / PEKA
0
200
400
600
800
1000
HNRNPL (HepG2)
SRSF1 (K562)
IGF2BP1 (K562)
IGF2BP1 (HepG2)
FXR1 (K562)
Clippy
NarrowPeaks
PEKA
STREME
d)
Clippy outperforms NarrowPeaks in PEKA
HNRNPC (HepG2)
HNRNPC (K562)
HNRNPA1 (HepG2)
SRSF7 (HepG2)
CPEB4 (K562)

Fig S4 | Influence of size-matched input controls on motif discovery in PEKA and STREME.

a) K-mer logo shows sequence features of 20 most enriched 5-mers for HNRNPC as ranked by RBNS. **b)** Lineplots show average motif coverage of 20 most enriched 5-mers for HNRNPC (in RBNS) around crosslink sites for HNRNPC eCLIP and SMInput. The top plot shows HNRNPC eCLIP and SMInput in HepG2 cells and the bottom plot shows HNRNPC eCLIP and SMInput K562 cell line. **c, d)** Comparison of motif discovery by PEKA and STREME using either Clippy peaks or narrowPeaks, for five eCLIPs where the use of narrowPeaks c) increased or d) decreased recall the most, when compared to PEKA run with Clippy peaks (results shown in Figure 3). Pointplot shows the median ranking of significantly enriched 5-mers ($p < 0.05$) for each combination of motif discovery method (PEKA / STREME) and peaks (Clippy / narrowPeaks) in the corresponding *in vitro* dataset (Methods). Vertical lines represent the standard deviation. Heatmaps above the pointplot show nucleotide frequencies in shuffled peak sequences that were used as background in STREME with Clippy peaks (top) or narrowPeaks (bottom).

Fig S5

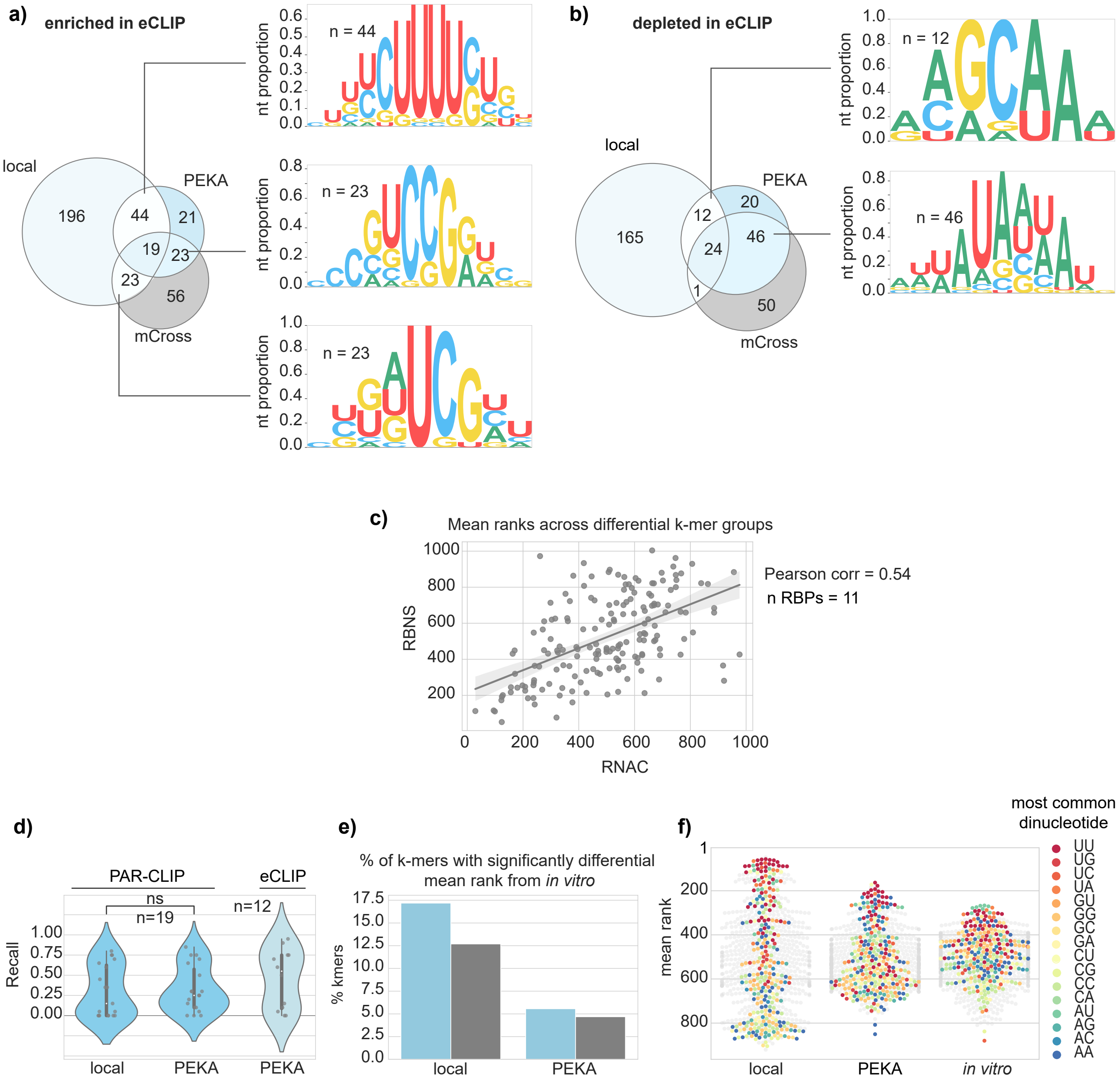
a)
enriched in eCLIP
local
PEKA
mCross
196
44
21
19
23
23
56
n = 44
n = 23
n = 23
nt proportion
b)
depleted in eCLIP
local
PEKA
mCross
165
12
20
24
46
1
50
n = 12
n = 46
nt proportion
c)
Mean ranks across differential k-mer groups
RBNS
RNAC
Pearson corr = 0.54
n RBPs = 11
d)
PAR-CLIP
eCLIP
ns
n=19
n=12
Recall
local
PEKA
PEKA
e)
% of k-mers with significantly differential mean rank from in vitro
% kmers
local
PEKA
f)
most common dinucleotide
UU
UG
UC
UA
GU
GG
GC
GA
CU
CG
CC
CA
AU
AG
AC
AA
mean rank
local
PEKA
in vitro

Fig S5 | Differential enrichment of motif groups in eCLIP and PAR-CLIP relative to *in vitro* methods.

a,b) Venn diagrams show the overlap across groups of 5-mers with significant difference in ranking between *in vitro* and eCLIP, analysed with PEKA, raw mCross, and 'local' approach. K-mer groups that were differentially enriched in eCLIP, relative to *in vitro*, are in panel a) and groups of k-mers that were depleted in eCLIP, relative to *in vitro*, are shown in panel b). K-mer logos show characteristics of k-mers contained in each group. Here, we show k-mer logos for groups of k-mers that were not presented in Figure 4 d, e, except for the overlap between the k-mers depleted in 'local' approach and raw mCross, which encompasses only 1 k-mer. **c)** Scatterplot shows mean k-mer ranks across differentially ranked motif groups for 11 RBPs that had RNAC and RBNS data available, and also have an overlapping eCLIP dataset. The line represents the regression model fitted to datapoints and the shaded area around the regression line corresponds to the 95% confidence interval for the regression estimate. **d)** Blue violin plots on the left compare recall achieved by PEKA or 'local' approach across 19 PAR-CLIP datasets for which *in vitro* data was available. Lightblue violin plot on the right shows recall distribution for 12 RBPs in eCLIP that overlap with PAR-CLIP RBPs (in cases of RBPs where eCLIPs were available for both cell lines, the dataset with higher recall is shown). **e)** Percentage of k-mers with significantly differential ranking (Welch's t-test $p < 0.01$ and a fold-change greater than 1.5 or less than 0.66) between PAR-CLIP and *in vitro* for PEKA and 'local' approach. Differential ranking was assessed on 19 PAR-CLIP datasets for which *in vitro* data was available. **f)** Swarmplot shows mean k-mer ranks across RBPs ($n = 19$) in PEKA and 'local' approach. K-mers which contain two or more of the same dinucleotide in their sequence are colored by their most common dinucleotide and other k-mers are shown in gray.

Fig S6

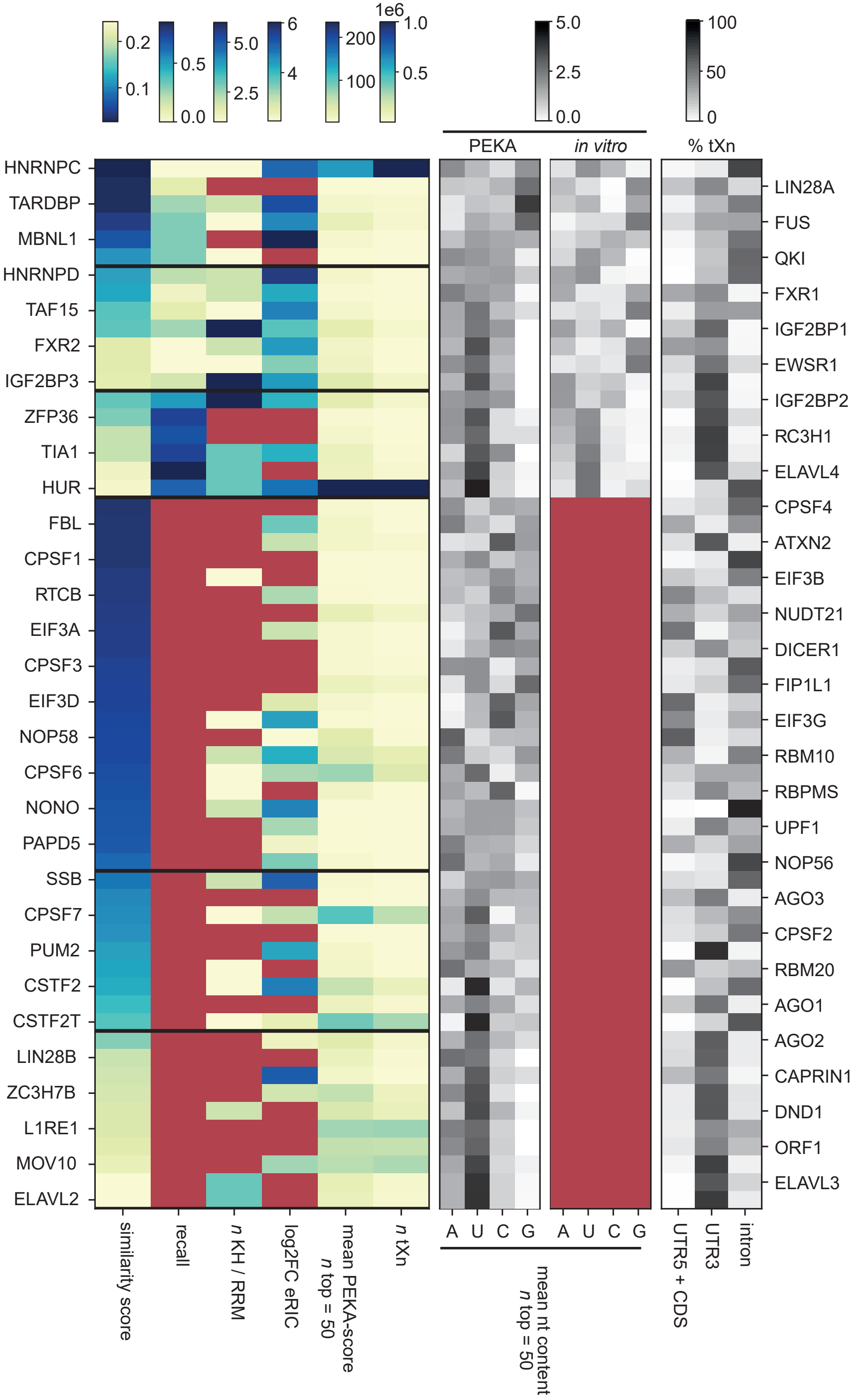

0.2
0.1
0.5
0.0
5.0
2.5
6
4
200
100
1e6
1.0
0.5
5.0
2.5
0.0
100
50
0
PEKA
in vitro
% tXn
HNRNPC
TARDBP
MBNL1
HNRNPD
TAF15
FXR2
IGF2BP3
ZFP36
TIA1
HUR
FBL
CPSF1
RTCB
EIF3A
CPSF3
EIF3D
NOP58
CPSF6
NONO
PAPD5
SSB
CPSF7
PUM2
CSTF2
CSTF2T
LIN28B
ZC3H7B
L1RE1
MOV10
ELAVL2
LIN28A
FUS
QKI
FXR1
IGF2BP1
EWSR1
IGF2BP2
RC3H1
ELAVL4
CPSF4
ATXN2
EIF3B
NUDT21
DICER1
FIP1L1
EIF3G
RBM10
RBPMS
UPF1
NOP56
AGO3
CPSF2
RBM20
AGO1
AGO2
CAPRIN1
DND1
ORF1
ELAVL3
similarity score
recall
n KH / RRM
log2FC eRIC
mean PEKA-score n top = 50
n tXn
A U C G
A U C G
mean nt content n top = 50
UTR5 + CDS
UTR3
intron

Fig S6 | Expected specificity of PAR-CLIP datasets with respect to various RBP features.

Heatmap on the left shows 61 PAR-CLIP datasets, clustered based on their similarity scores and recall into 6 clusters. For each dataset the heatmap also shows its enrichment in the mRNA interactome proteomics (log2FC eRIC), the number of KH or RRM in the RBP, the average PEKA-score across the top 50 ranked k-mers, and the number of thresholded crosslinks in the 'protein-coding gene' region. Values shown on this heatmap are available in Additional file 9. Grayscale heatmaps from left to right show mean nucleotide content across the top 50 ranked 5-mers for each dataset in PEKA (left) and *in vitro* data (middle) and % of thresholded crosslinks derived from each transcript region (right).

Bibliography

1. Feng H, Bao S, Rahman MA, Weyn-Vanhentenryck SM, Khan A, Wong J, et al. Modeling RNA-Binding Protein Specificity In Vivo by Precisely Registering Protein-RNA Crosslink Sites. Mol Cell. 2019;74:1189–204.e6.

2. Mahony S, Benos PV. STAMP: a web tool for exploring DNA-binding motif similarities. Nucleic Acids Res. 2007;35:W253–8.
