# Supplementary material for: Positional motif analysis reveals the extent of specificity of protein-RNA interactions observed by CLIP
Source: Genome Biol. 2022 Sep 9;23:191. doi: 10.1186/s13059-022-02755-2 (PMC9461102; doi:10.1186/s13059-022-02755-2)
Supplement: Supplementary file 11 — Additional file 11. Review history. [file 13059_2022_2755_MOESM11_ESM.docx]

Review History

**First round of review**

**Reviewer 1**

**Were you able to assess all statistics in the manuscript, including the appropriateness of statistical tests used?**

Yes

**Comments to author:**

In this manuscript, Kuret and colleagues developed a new method to better assess the specificity of motif enrichment in CLIP variant datas compared to in vitro and existing methods. The work is very elegant using appropriate methods, and presented in an easy understandable way. The authors went point by point to prove the validity and advantages of the PEKA method. the conclusions are adequate and I highly recommend the publication of this manuscript simply because it will be of great help to the RNA community to better assess the motif specificity of each RBP.

Below you can find the minor comments that will help to improve the understanding of the paper for non computational audience:

1- figure 1b Can PEKA be used with IgG or no antibody samples?

2- figure 1d Could this heat map be produced for TIA iCLIP? to reinforce or eliminate the hypothesis of the effect of a co purified protein during the experimental set

3-figure 2: how many proteins have higher recall in iclip vs eclip? could this reflect the specificity of iclip vs eclip?

4-figure 3b change colours of violin plot U and C (confusing with the colours of paraclu and NP)

5- figure 4d-e show the significance (by notches on the box plots)

6- Figure 5 is the enrichment due to the environmental sequence. are the profiles different in iCLIP compared to eCLIP (regional cross linking profiles)?

**Reviewer 2**

**Were you able to assess all statistics in the manuscript, including the appropriateness of statistical tests used?**

N/A

**Comments to author:**

The manuscript by Kuret et al., presents an elegant tool named PEKA (positionally-enriched k-mer analysis) to detect enriched motifs in CLIP data. The tool is nicely designed to detect motifs in a position specific manner while minimizing the influence of technical and regional biases by normalizing the occurrences of k-mers at around high confidence crosslink sites to their occurrences in out-of-peak crosslinked sites (considered as background). The method was benchmarked on in-vitro data and exhibited similar performance to a relatively recent published tool for Modeling RNA-Binding Protein Specificity (mCross). The authors further test PEKA on in-vivo data, focusing specifically on eCLIP datasets. Based on their analyses they conclude that there are strong technical biases and contamination in eCLIP data that question the reliability of the technology. They finally propose that PEKA can be used for overall assessing reliability of CLIP data. Overall, while there are many tools available for motif detection I find that the unique features of PEKA can help extract important information from in-vivo RNA-binding data and contribute to study RNA-binding specificities. However, I have major concerns regarding the biased analyses that were conducted in the study and the conclusions and interpretations of the results.

Below are my specific comments

1. One of the unique features of PEKA is that the k-mer occurrences at a given position within the proximal regions around the crosslinked sites are normalized to the k-mer occurrences in the distal regions. While this approach may be helpful to reduce biases, I expect that for some RBPs this may lead to miss detection of biologically relevant motifs. This could be specifically true for RBPs that tend to bind in clusters, where the extended environment around the binding sites may have characteristic features that differ significantly from background sequences. The authors do show that PEKA was able to discover motifs that are broadly enriched around crosslink sites in the case of one protein, TDP-43. However, to confirm that the approach does not affect motif detection in RBPs that bind RNA in a similar manner it is critical to conduct the analyses for a large set of RBPs with and without the regional normalization. In addition it would be highly informative to present the results on a range of window sizes around the crosslinked site (proximal region).

2. The authors describe results of PEKA analysis conducted on eCLIP dataset for the TIA RBP, as an example to demonstrate that PEKA can detect RBPs with different modes of binding. They further argue that the additional G-rich motif that they detect may result from other co-purified motifs. However, if the G-rich motif indeed does result from other co-purified RBPs I would expect that the normalization to background would eliminate them, as the case when running mCross on this dataset (when using normalized scores).

This examples raises questions regarding the interpretation of PEKA results when running it on individual datasets, and whether it is possible to distinguish between biological relevant motifs and artifacts without conducting a meta-analysis .

Can the author provide other examples of RBPs that were detected by PEKA to have different binding specificity and have been experimentally confirmed as such?.

3. The authors benchmark PEKA against 28 different RBPs for which results are available from in-vitro (RNBS and RNAC) data and compare PEKA results to the published tool mCross. The conclusion from this analysis is that PEKA performs very similar to mCross, however, I was not able to find the detailed results of these analyses. Based on their conclusion can the author elaborate on what is the added value of PEKA over mCross?.

4. While the author present PEKA as a useful tool to assess CLIP data, it seems that the main emphasis of the study is to test the reliability of eCLIP. Indeed most of the RNA-binding data available in the public domain is from eCLIP, still there is a large amount of data available from other CLIP technologies (iCLIP and PAR-CLIP). It is expected that the authors would present a more comprehensive analysis of a balanced dataset. To my knowledge there are many more RBPs for which in-vivo binding data is available from (at least) two different technologies for which in-vitro data is also available, either from RBNS or RNAC. Conducting such comparisons will allow better assessing the method as well as the biases in the different CLIP technologies.

5. The analysis of eCLIP results with and without the size matched input (SMI)-correction is an important test and is specifically useful for the PEKA users. Nevertheless, given that the results presented strongly hint to a bias in the SMI correction in eCLIP it is essential that the author will conduct a similar analysis using a different enrichment analysis tool (such as mCross, which is used as a reference throughout the manuscript) to confirm that the results are not specific to PEKA.

6. From the analysis comparing PEKA and mCross to a simple 'local' approach the author conclude that eCLIP has technical biases and that correction to background is essential. This is a well-established phenomenon and in the basic for any motif enrichment tool. To evaluate the added value of PEKA in detecting technical biases in CLIP the authors should present a similar analysis conducted on other CLIP datasets generated from different technologies, as would be expected based on the title of the paragraph " Insights into technical biases of CLIP".

7. In PEKA reliability assessment the author put a strong emphasis on the similarity score, specifically the similarity between the results (top k-mers) from the in-vivo experiments to those from in-vitro data (when available). In-vitro experiments capture the intrinsic binding preferences of the proteins and thus comparison to motifs from in-vitro data indeed should be one of the parameters to assess the data. Nevertheless one should be very careful when interpreting the results, as for some RBPs in-vitro data would not be the right "gold standard". While the authors do refer to this in the manuscript they should emphasize this point in the discussion section, where they elaborate on the low reliability of the eCLIP results.

8. The observation that many eCLIP dataset share high motif preference can certainly indicate that there are biases/contaminations in the data (possibly due to the fact that these proteins do not crosslink well). Furthermore, it is probable that proteins for which orthogonal in-vitro binding data is not available have unique features that likely results from their weaker binding specificities. However, this does not mean that the motif detected for these proteins are necessarily artifacts. To test this, it would be essential to conduct the same systematic analysis for large sets of datasets generated by the other CLIP technologies focusing on proteins that do not contain classical RBDs and likely have very different mode of binding than conventional RBPs.

Minor comments

1. The details of PEKA are clearly described in Figure 1 and in the method section, however, several steps in the process that strongly influence the results, such as the how was the cDNA threshold (70%) determined, seems to be chosen arbitrary and not clearly explained.

2. In the comparison between eCLIP and iCLIP the authors compare results from eCLIP for TIA1 RBP to the iCLIP results for TIAL1 (TIA like). Are these the same proteins? This should be explained. In addition it should be clear in the text that iCLIP data is from TIAL1 and not TIA (line 232).

3. It is unclear why the author chose the eRIC database as their reference for the RBPome. There are many other RIC datasets and their combined results are available in several review papers. In this case it may be reasonable to focus on RIC data from the same cell line (for k562 such data is available from Conrad et al.., (2016). Accordingly, the statistics presented in the manuscript and data presented in Figure 6 should be corrected.

**Authors Response**

**Point-by-point responses to the reviewers’ comments:**

*We thank the reviewers for their time and effort and their detailed suggestions, which helped us to improve the presentation of results, clarify the methods and conclusions in the text and add several new analyses, including the analysis of the large PAR-CLIP data resource. This led to major revisions of Figures 4 and 6, and Supplementary figures 1, 2, 4 and 6. We made effort to thoroughly address all the suggestions, and we believe the manuscript is now more thorough and accessible to the broad readership of Genome biology. We will of course be happy to make adjustments if reviewers will have further suggestions.*

Comments from Reviewer 1

Comment 1: figure 1b Can PEKA be used with IgG or no antibody samples?

*Response: Yes, PEKA can be run on any type of nucleotide-resolution quantitative genomic data (even non-CLIP), so it can in principle be run on individual IgG, or no antibody CLIP data. However, it’s not recommended, as PEKA will only be able to generate results when a library contains sufficient complexity of cDNAs and peaks containing positions with cDNA counts>1. This should not be the case for individual IgG or no antibody samples if properly implemented, as they should not yield much signal. Nevertheless, if the control data do produce complex libraries, PEKA can be run on (for example, input control data such as eCLIP SMInput), PEKA might identify motifs enriched in such control datasets, and this can then be compared with motifs enriched in CLIP datasets of specific RBPs to identify candidate RBPs if they are very strong primary contributors to the control signal.*

Comment 2: figure 1d Could this heat map be produced for TIA iCLIP? to reinforce or eliminate the hypothesis of the effect of a co-purified protein during the experimental set

*Response: We assumed that the reviewer referred to Figure 1g, and we have indeed now placed the heatmap with TIA1 eCLIP into Fig 1g, and TIA1 iCLIP and PAR-CLIP into Supplementary figure 1. This shows more directly that G-rich motifs are highly enriched only in TIA1 eCLIP.*

Comment 3: figure 2: how many proteins have higher recall in iclip vs eclip? could this reflect the specificity of iclip vs eclip?

*Response: We agree with the reviewer, and for this purpose we analysed the large multi-protein PAR-CLIP resource fully in the same way as eCLIP, and we present it in Supplementary figure 5 (in the manner analogous to the Fig 6 for eCLIP), which leads to additional insights including: 1) For both methods, U-binders tend to have highest recall, 2) Datasets that tend to have highest motif similarity to each other have enrichment of G-rich motifs for eCLIP, and U-rich motifs for PAR-CLIP, 3) Presence of RRM/KH domains correlates to high recall in eCLIP, but not in PAR-CLIP, 4) RBPs that generate unique motif enrichments (low similarity index) tend to have high numbers of RRM/KH domains in eCLIP, but no such correlation is seen for PAR-CLIP (where instead we see a slightly reverse trend, with most unique data for RBPs that lack KH/RRM domains). For now, eCLIP and PAR-CLIP are the only variants that have been used to produce large multi-protein data resources, which are best suited to comparative analyses.*

*In the case of iCLIP, we have limited ourselves to data produced from the Ule lab to avoid cross-lab variations and the curation problems encountered when processing data from other labs. Nevertheless, we produced an online platform (https://imaps.goodwright.com/) that now allows all labs to curate, upload and process their data freely (including running PEKA) and make it publicly available. Our plan is that as such new data accumulate, their PEKA outputs will be automatically integrated into the cross-RBP and cross-method comparisons at https://imaps.goodwright.com/apps/peka/, and thus over the near future the community will be able to increasingly gain better comparative insights. If the reviewer has particular iCLIP (or other) datasets in mind that they would like to see included in the paper, we would be happy to add them to our analysis.*

Comment 4: figure 3b change colours of violin plot U and C (confusing with the colours of paraclu and NP)

*Response: This is now modified.*

Comment 5: figure 4d-e show the significance (by notches on the box plots)

*Response: Thanks for the suggestion, now implemented.*

Comment 6: Figure 5 is the enrichment due to the environmental sequence. are the profiles different in iCLIP compared to eCLIP (regional cross linking profiles)?

*Response: In figure 5 we show that proteins that preferentially bind a certain region gravitate towards certain motif preferences, so indeed, regional profiles do affect motif enrichment. The reasons for these regional imbalances are probably complex and would require further cross-method comparisons for full understanding. For example, we see most enrichment of U-rich k-mers in 3′-UTR for both eCLIP and PAR-CLIP, which aligns with the generally highest prevalence of AREs and related motifs in 3′-UTRs. Conversely, we see strongest enrichment of G-rich motifs in introns, but only for eCLIP, so here it may reflect common co-purification of a specific contaminating RBP that binds G-rich motifs, rather than a generic regional motif bias.*

*The reviewer is also asking about the % tXn across regions (intron / UTR / CDS)? We show this analysis in Figure 2, where we show that PAR-CLIP and iCLIP often obtain higher % in 3′-UTRs compared to eCLIP. We comment on both aspects in the manuscript.*

Comments from Reviewer 2

Comment 1: One of the unique features of PEKA is that the k-mer occurrences at a given position within the proximal regions around the crosslinked sites are normalized to the k-mer occurrences in the distal regions. While this approach may be helpful to reduce biases, I expect that for some RBPs this may lead to miss detection of biologically relevant motifs. This could be specifically true for RBPs that tend to bind in clusters, where the extended environment around the binding sites may have characteristic features that differ significantly from background sequences. The authors do show that PEKA was able to discover motifs that are broadly enriched around crosslink sites in the case of one protein, TDP-43. However, to confirm that the approach does not affect motif detection in RBPs that bind RNA in a similar manner it is critical to conduct the analyses for a large set of RBPs with and without the regional normalization. In addition, it would be highly informative to present the results on a range of window sizes around the crosslinked site (proximal region).

*Response: We thank the reviewer for this suggestion, which we address (as explained below), but first to clarify that PEKA controls for technical biases by normalising with the intrinsic background (i.e., the relative occurrence around oXn), and not by the occurrence with distal regions. The advantage of normalising with the intrinsic background is that it should decrease both the regional biases as well as biases at crosslink sites that are present for technical reasons (such as crosslink biases, ligation biases, etc.) - whereas normalisation by distal regions would normalise only for regional biases. PEKA only performs normalisation with distal region to define relevant positions around crosslink site that are used to calculate motif enrichment (thereby to identify positions where occurrences of k-mers are above the background level), which only indirectly affects PEKA-scores via selection of relevant positions. However, PEKA also offers users an option to calculate enrichment by using all positions within a defined window around crosslink sites, in which case normalisation with distal regions would have no effect on PEKA-score. We now clarified this further in the text.*

*The normalisation by distal regions is done when we are visualising the enrichment of k-mers on heatmaps, but this is unrelated to PEKA-score. To address the reviewer’s request of evaluating the two normalisation approaches, we now compare in Figure 4 the motif ranks obtained by PEKA-scores with those obtained when we only normalise with distal regions (‘the local approach’), and with ranks obtained by mCross that uses a more complex algorithm based on normalisation with distal regions. We find that motif ranks obtained by PEKA-scores generally agree better with those from in vitro data (i.e., higher recall, Figure 4a), and have less sequence biases (Figure 4c) than ‘the local approach’. Moreover, we show that motifs that are most highly enriched with the local approach tend to have strong U-bias (see ‘unique local’ in Figure 4d), which is expected given that the local approach searches for motifs enriched at -3..3 nts around crosslink sites. In contrast, motifs most highly enriched in PEKA as compared to other approaches (see ‘unique PEKA’ in Figure 4d) tend to be broadly enriched around crosslinks and have a bias for C (a poorly crosslinking nucleotide). Importantly, when a motif is highly enriched for a specific RBP, PEKA will identify it as top-ranking regardless of its motif class or positional pattern as shown by the examples in Figure 4d,e.*

*We also show that PEKA achieves similar performance to mCross when looking at a recovery of in vitro motifs (Suppl. Figure 3a) and that both methods result in similar sequence biases, with exception of minor differences (PEKA tends to enrich U-rich k-mers more and mCross has a tendency to deplete AU-rich k-mers) (Figure 4 f,g). This indicates that both methods of normalisation can efficiently detect relevant motifs, but due to specifics of each method, each method might have some advantages for specific RBPs, which likely will become clearer with further studies.*

Comment 2: The authors describe results of PEKA analysis conducted on eCLIP dataset for the TIA RBP, as an example to demonstrate that PEKA can detect RBPs with different modes of binding. They further argue that the additional G-rich motif that they detect may result from other co-purified motifs. However, if the G-rich motif indeed does result from other co-purified RBPs I would expect that the normalization to background would eliminate them, as the case when running mCross on this dataset (when using normalized scores). This examples raises questions regarding the interpretation of PEKA results when running it on individual datasets, and whether it is possible to distinguish between biological relevant motifs and artifacts without conducting a meta-analysis. Can the author provide other examples of RBPs that were detected by PEKA to have different binding specificity and have been experimentally confirmed as such?

*Response: As we describe in the manuscript, PEKA aims to normalise data against its own intrinsic background (i.e., the relative occurrence around oXn, and therefore it can’t distinguish whether enriched motifs represent binding of the protein that was meant to be IPed or another co-purified RBP. However, a meta-analysis across large numbers of datasets can yield insights into features that might reflect more generic technical biases or common contaminants. In Figures 5 and 6 we show that G-rich motifs are commonly enriched across eCLIP datasets, whereas this is not the case across PAR-CLIP datasets as shown in Suppl. figure 6, where instead a stronger prevalence of U-rich motifs is seen.*

*In Supplementary figure 3c, we also compare enriched motifs between PEKA and raw and normalised mCross for TIA1 eCLIP and show that the G-rich motifs are enriched in raw mCross scores, similarly to PEKA, but are reduced in the normalised mCross scores. These normalised mCross scores are derived by normalising with median z-scores of motifs across all eCLIP datasets after they have been individually processed with mCross, and thus could not be derived if each dataset was analysed individually. Thus, we achieve the same purpose as the mCross study has done by normalising across all datasets, but in our case, we don’t just remove the commonly enriched motifs, but rather visualise and analyse such common enrichment, as it can help in the interpretation of eCLIP datasets. We believe this is a more informative approach, because commonly enriched motifs don’t necessarily represent background in all cases - they likely represent valid binding motifs in a subset of experiments, and it is important to be aware of them as a feature to be considered when interpreting other eCLIP experiments.*

“Can the author provide other examples of RBPs that were detected by PEKA to have different binding specificity and have been experimentally confirmed as such?”

*Response: To address this point we now added the relative occurrence heatmaps for TDP-43, LIN28B and QKI in Supplementary figure 2. TDP-43 shows strongest binding to UG-repeat, but also binds GUAU motifs and UGAA motifs as demonstrated previously by iCLIP experiments that showed their distinct binding preferences to mutant variants of TDP-43 (Hallegger et al., 2021). Indeed, we show that PEKA successfully identifies these motif groups from eCLIP data. LIN28 is known to bind (U)GAU k-mer, as well as GGAG-like sequences (Ustianenko et al., 2018), which are also both recovered among the top 20 enriched k-mers. Finally, QKI binds bipartite motifs, which are identified and visualised with PEKA, showing a peak of enrichment both upstream and downstream of crosslink sites (Galarneau and Richard, 2005; Conn et al., 2015). Such bimodal enrichment patterns are not apparent from the PWM-based visualisations provided by mCross (see for example https://zhanglab.c2b2.columbia.edu/mCrossBase/rbp.php?id=HepG2.QKI). We would also wish to remind the reviewer that such plots are available for all proteins at our web platform (see for example* [*https://imaps.goodwright.com/apps/peka?rbp=HepG2-QKI*](https://imaps.goodwright.com/apps/peka?rbp=HepG2-QKI)*).*

Comment 3: The authors benchmark PEKA against 28 different RBPs for which results are available from in-vitro (RNBS and RNAC) data and compare PEKA results to the published tool mCross. The conclusion from this analysis is that PEKA performs very similar to mCross, however, I was not able to find the detailed results of these analyses. Based on their conclusion can the author elaborate on what is the added value of PEKA over mCross?

*Response: We have now added detailed values of recall analysis which compares the capacity of PEKA and raw mCrossto recover highly ranked in vitro k-mers from eCLIP data in the Additional file 8 (Suppl. Figure 3a). Moreover, we added qualitative and quantitative analysis of motif groups that show differential enrichment between in vitro data and eCLIP analysed with either PEKA, raw mCross and ‘local’ approach in Figure 4 and Suppl. Figure 5a,b. While the recall of both approaches is comparable (Supplementary figure 3a), there are several advantages of PEKA:*

*It is able analyse a larger set of RBPs. 16 RBPs with available orthogonal in vitro data were excluded from mCross (shown in Supplementary figure 3b) because (according to the mCross method section), they did not yield sufficient number of significantly enriched k-mers and/or showed low concordance between replicates in terms of the enriched 7-mers. However, we suspect that in several cases low concordance in enriched motifs and low number of significantly enriched k-mers was caused by normalisation, performed in mCross, which decreases z-scores of k-mers commonly enriched in these 16 eCLIP experiments, namely G-rich k-mers and U-rich k-mers, as exemplified by FUS and HNRNPC, respectively. In PEKA, most of these RBPs yield excellent recall, so if these RBPs are considered for the comparison, we could claim that PEKA overall performs better.*

*The results are differently visualised - mCross aims to generate metamotifs (weblogos), while PEKA outputs k-mer clusters without generating logos. This enables PEKA to visualise the complex and mechanistically-relevant enrichment patterns (compare the bimodal pattern visible in https://imaps.goodwright.com/apps/peka?rbp=HepG2-QKI but not in https://zhanglab.c2b2.columbia.edu/mCrossBase/rbp.php?id=HepG2.QKI). Moreover, PEKA is able to discover GGAG-type motifs bound by the zinc knuckle domain (ZKD) of LIN28B which were not reported by mCross (compare (https://imaps.goodwright.com/apps/peka?rbp=K562-LIN28B with https://zhanglab.c2b2.columbia.edu/mCrossBase/rbp.php?id=K562.LIN28B)(Ustianenko et al., 2018), which highlights the strength of PEKA for nuanced motif discovery. Finally, the k-mer-based output of PEKA is also more compatible with further cross-RBP comparative visualisations of motifs.*

*Unlike mCross, PEKA performs regional analysis and visualisation of enriched motifs, so it can distinguish between motifs specific to a particular genomic region for the investigated RBP.*

*PEKA code is available open-source via GitHub (https://github.com/ulelab/peka) and Bioconda (https://anaconda.org/bioconda/peka) in a very accessible and user-friendly manner, which allows users to adjust settings, whereas the mCross code is not available. PEKA is also integrated into the iMaps platform for reproducible analysis of CLIP data (https://imaps.goodwright.com/).*

*We now clarify these advantages also in the manuscript.*

Comment 4: While the author present PEKA as a useful tool to assess CLIP data, it seems that the main emphasis of the study is to test the reliability of eCLIP. Indeed most of the RNA-binding data available in the public domain is from eCLIP, still there is a large amount of data available from other CLIP technologies (iCLIP and PAR-CLIP). It is expected that the authors would present a more comprehensive analysis of a balanced dataset. To my knowledge, there are many more RBPs for which in-vivo binding data is available from (at least) two different technologies for which in-vitro data is also available, either from RBNS or RNAC. Conducting such comparisons will allow better assessing the method as well as the biases in the different CLIP technologies.

*Response: We agree with the reviewer, and for this purpose, we analysed 61 PAR-CLIP datasets form several multi-protein resources fully in the same way as eCLIP, and we present it in Supplementary figure 6 (in the manner analogous to the Figure 6 for eCLIP), which leads to additional insights including: 1) For both methods, U-binders tend to have highest recall, 2) Datasets that tend to have highest motif similarity to each other have enrichment of G-rich motifs for eCLIP, and U-rich motifs for PAR-CLIP, 3) Presence of RRM/KH domains correlates to high recall in eCLIP, but not in PAR-CLIP, 4) RBPs that generate unique motif enrichments (low similarity index) tend to have high numbers of RRM/KH domains in eCLIP, but no such correlation is seen for PAR-CLIP (where instead we see a slightly reverse trend, with most unique data for RBPs that lack KH/RRM domains). For now, eCLIP and PAR-CLIP are the only variants that have been used to produce large multi-protein data resources, which are best suited to comparative analyses.*

*In the case of iCLIP, we have limited ourselves to data produced from the Ule lab to avoid cross-lab variations and the curation problems encountered when processing data from other labs. Nevertheless, we produced an online platform (https://imaps.goodwright.com/) that now allows all labs to curate, upload and process their data freely (including running PEKA) and make it publicly available. Our plan is that as such new data accumulate, their PEKA outputs will be automatically integrated into the cross-RBP and cross-method comparisons at https://imaps.goodwright.com/apps/peka/, and thus over the near future the community will be able to increasingly gain better comparative insights. If the reviewer has particular iCLIP (or other) datasets in mind that they would like to see included in the paper, we would be happy to add them to our analysis. Currently included iCLIP and PAR-CLIP datasets are listed in Additional file 3.*

Comment 5: The analysis of eCLIP results with and without the size-matched input (SMI)-correction is an important test and is specifically useful for PEKA users. Nevertheless, given that the results presented strongly hint to a bias in the SMI correction in eCLIP it is essential that the author will conduct a similar analysis using a different enrichment analysis tool (such as mCross, which is used as a reference throughout the manuscript) to confirm that the results are not specific to PEKA.

*Response: The reviewer suggests that we use mCross to examine the importance of size-matched input (SMI)-correction, however this would be challenging. The mCross publication didn’t evaluate SMInput, and the mCross code is not accessible, thus we can’t use this approach to examine the value of using SMInputs. Instead, we now examined in more depth the 10 eCLIP datasets where recall was most impacted (positively or negatively) when using PEKA with Clippy peaks that do not use SMInput, or with narrowPeaks that do use the SMInput for normalisation (Figure 3a). For this, we used STREME (Bailey, 2021), a commonly used tool to find enriched motifs from CLIP data, and provided it either with sequences from Clippy or narrowPeaks. We then compared the ranking of significantly enriched k-mers in PEKA or STREME (motifs with p < 0.05) with the corresponding in vitro data. STREME uses shuffled peak sequences as background, so its analysis approach is different from the one used in PEKA, thus providing an independent approach to investigate the effects of using SMInput for analysis of eCLIP data. This showed that in the cases of RBPs where PEKA analysis of narrowPeaks gave better ranking than Clippy peaks, STREME analysis also achieved better ranking with narrowPeaks for all datasets (Suppl. Figure 4c, Additional file 1). Furthermore, in the cases of RBPs where PEKA analysis of Clippy peaks gave better ranking than narrowPeaks, STREME analysis also recovered motifs with either higher or similar ranking for Clippy peaks compared to narrowPeaks (Suppl. Figure 4d, Additional file 1). This indicates that the lack of general motif improvement from narrowPeaks as compared to Clippy peaks in PEKA is reliable, since the alternative motif analysis approach follows the same trends. Thus, the comparison of narrowPeaks with Clippy peaks with STREME confirms the findings from PEKA that the use of SMI correction does not seem to yield systematic improvements in the specificity of CLIP data.*

Comment 6: From the analysis comparing PEKA and mCross to a simple 'local' approach the author conclude that eCLIP has technical biases and that correction to background is essential. This is a well-established phenomenon and in the basic for any motif enrichment tool. To evaluate the added value of PEKA in detecting technical biases in CLIP the authors should present a similar analysis conducted on other CLIP datasets generated from different technologies, as would be expected based on the title of the paragraph " Insights into technical biases of CLIP".

*Response: Again, we agree with the reviewer, and for this purpose we analysed 61 PAR-CLIP datasets fully in the same way as eCLIP, and we present it in Supplementary figure 6 (in the manner analogous to the Figure 6 for eCLIP), which leads to additional insights including: 1) For both methods, U-binders tend to have highest recall, 2) Datasets that tend to have highest motif similarity to each other have enrichment of G-rich motifs for eCLIP, and U-rich motifs for PAR-CLIP, 3) Presence of RRM/KH domains correlates to high recall in eCLIP, but not in PAR-CLIP, 4) RBPs that generate unique motif enrichments (low similarity index) tend to have high numbers of RRM/KH domains in eCLIP, but no such correlation is seen for PAR-CLIP (where instead we see a slightly reverse trend, with most unique data for RBPs that lack KH/RRM domains). For now, eCLIP and PAR-CLIP are the only variants that have been used to produce large multi-protein data resources, which are best suited to comparative analyses.*

Comment 7: In PEKA reliability assessment the author put a strong emphasis on the similarity score, specifically the similarity between the results (top k-mers) from the in-vivo experiments to those from in-vitro data (when available). In-vitro experiments capture the intrinsic binding preferences of the proteins and thus comparison to motifs from in-vitro data indeed should be one of the parameters to assess the data. Nevertheless one should be very careful when interpreting the results, as for some RBPs in-vitro data would not be the right "gold standard". While the authors do refer to this in the manuscript they should emphasize this point in the discussion section, where they elaborate on the low reliability of the eCLIP results.

*Response: This is an excellent suggestion, and we now emphasized further in the discussion that in many cases the motifs derived from CLIP will better represent RBP specificity than in vitro data. To better understand the in vivo specificity, comparisons across multiple in vivo methods and datasets are valuable, and as more data becomes available, we certainly will be able to gain increased certainty into the biological importance of in vivo specificity that differs from in vitro data.*

Comment 8: The observation that many eCLIP dataset share high motif preference can certainly indicate that there are biases/contaminations in the data (possibly due to the fact that these proteins do not crosslink well). Furthermore, it is probable that proteins for which orthogonal in-vitro binding data is not available have unique features that likely results from their weaker binding specificities. However, this does not mean that the motif detected for these proteins are necessarily artifacts. To test this, it would be essential to conduct the same systematic analysis for large sets of datasets generated by the other CLIP technologies focusing on proteins that do not contain classical RBDs and likely have very different mode of binding than conventional RBPs.

*Response: We agree with the reviewer, and for this purpose we analysed the large multi-protein PAR-CLIP resource fully in the same way as eCLIP, and we present it in Supplementary figure 5 (in the manner analogous to the Fig 6 for eCLIP). This showed that RBPs that generate unique motif enrichments (low similarity index) tend to have high numbers of RRM/KH domains in eCLIP, but no such correlation is seen for PAR-CLIP (where instead we see a slightly reverse trend, with most unique data for RBPs that lack KH/RRM domains). This perhaps argues further towards the possibility of higher contaminating signal in eCLIP (but not PAR-CLIP) for RBPs that lack KH/RRM domains (and thus likely less efficiently crosslink to RNA). Nevertheless, eCLIP and PAR-CLIP are the only variants that have been used to produce large multi-protein data resources, and further comparisons are needed before one can make solid conclusions, therefore we now highlighted the alternative interpretations offered by the reviewer also in our discussion.*

*We should also add that a useful insight is gained also by comparing the eCLIP data for PUM1 and PUM2, which are known to have similar binding, but only PUM2 identifies the expected motifs and has high recall (https://imaps.goodwright.com/apps/peka?rbp=K562-PUM2), whereas PUM1 exhibits the G-rich motifs (https://imaps.goodwright.com/apps/peka?rbp=K562-PUM1). This further suggests that G-rich motifs tend to be found enriched when an eCLIP experiment fails to identify a specific signal. It’s worth noting that the original eCLIP manuscript compared PUM2 eCLIP data with PUM1 RBNS and did not present analysis of the PUM1 eCLIP in the main figure (https://www.nature.com/articles/s41586-020-2077-3/figures/3).*

Minor comments

Minor comment 1: The details of PEKA are clearly described in Figure 1 and in the method section, however, several steps in the process that strongly influence the results, such as how was the cDNA threshold (70%) determined, seems to be chosen arbitrary and not clearly explained.

*Response: PEKA software allows users to determine the cDNA threshold, because this threshold largely depends on the cDNA complexity of the data. Currently available CLIP experiments (across variants) are dominated by crosslink positions identified with a single cDNA (i.e., score=1), and with the 70% threshold, in most peaks all positions with cDNA counts>1 are assigned as tXn. Increasing the threshold above 70% starts to decrease the number of assigned tXn sites, which can lead to less reliable results especially when datasets are small. Conversely, the users can set the threshold to 0, such that all crosslinks within peaks will be assigned as tXn and all out-of-peak xls as background. This is now more clearly described in methods.*

Minor comment 2: In the comparison between eCLIP and iCLIP the authors compare results from eCLIP for TIA1 RBP to the iCLIP results for TIAL1 (TIA like). Are these the same proteins? This should be explained. In addition it should be clear in the text that iCLIP data is from TIAL1 and not TIA (line 232).

*Response: We originally used TIAL1 iCLIP because its cDNA complexity was larger (it is more highly expressed in HEK cells and thus yields more signal), but we have now changed the analysis to TIA1 iCLIP since it produces virtually identical results.*

Minor comment 3: It is unclear why the author chose the eRIC database as their reference for the RBPome. There are many other RIC datasets and their combined results are available in several review papers. In this case it may be reasonable to focus on RIC data from the same cell line (for k562 such data is available from Conrad et al.., (2016). Accordingly, the statistics presented in the manuscript and data presented in Figure 6 should be corrected.

*Response: The reviewer refers to the manuscript that reports a nuclear interactome (https://pubmed.ncbi.nlm.nih.gov/27040163/). We prefer to use the interactome of whole cells, since eCLIP also used whole cell lysates. We chose eRIC because its data was well structured and quantitative information available to integrate with our study. The eRIC study reported log2FC over non-crosslinked control, important to account for some of the expression bias (several other papers only reported proteins that were found to be enriched, but not the extent of enrichment). It is also appropriate as a signature of binding to protein-coding transcripts as it is highly enriched for polyA-tailed RNAs, and we ran PEKA on the “whole gene” region of protein-coding genes.*

*References*

*Bailey, T. L. (2021) “STREME: Accurate and versatile sequence motif discovery,” Bioinformatics . doi: 10.1093/bioinformatics/btab203.*

*Conn, S. J. et al. (2015) “The RNA binding protein quaking regulates formation of circRNAs,” Cell, 160(6), pp. 1125–1134.*

*Galarneau, A. and Richard, S. (2005) “Target RNA motif and target mRNAs of the Quaking STAR protein,” Nature structural & molecular biology, 12(8), pp. 691–698.*

*Hallegger, M. et al. (2021) “TDP-43 condensation properties specify its RNA-binding and regulatory repertoire,” Cell. doi: 10.1016/j.cell.2021.07.018.*

*Ustianenko, D. et al. (2018) “LIN28 Selectively Modulates a Subclass of Let-7 MicroRNAs,” Molecular cell, 71(2), pp. 271-283.e5.*

**Second round of review**

**Reviewer 1**

I am satisfied with the answers provided. In my opinion the paper should be now accepted for publication.

**Reviewer 2**

All my concerns were addressed. The manuscript has significantly improved during revision.
